# Non-Coding RNAs in Human Breast Milk: A Systematic Review

**DOI:** 10.3389/fimmu.2021.725323

**Published:** 2021-09-01

**Authors:** Lina Tingö, Emelie Ahlberg, Lovisa Johansson, Sindre Andre Pedersen, Konika Chawla, Pål Sætrom, Erika Cione, Melanie Rae Simpson

**Affiliations:** ^1^Division of Inflammation and Infection, Department of Biomedical and Clinical Sciences, Linköping University, Linköping, Sweden; ^2^Örebro University Food and Health Programme, School of Medical Sciences, Örebro University, Örebro, Sweden; ^3^Division of Neurobiology, Department of Biomedical and Clinical Sciences, Linköping University, Linköping, Sweden; ^4^Library Section for Medicine and Health Sciences, NTNU Norwegian University of Science and Technology, Trondheim, Norway; ^5^Department of Clinical and Molecular Medicine, NTNU Norwegian University of Science and Technology, Trondheim, Norway; ^6^Bioinformatics Core Facility - BioCore, NTNU Norwegian University of Science and Technology, Trondheim, Norway; ^7^K.G. Jebsen Center for Genetic Epidemiology, NTNU Norwegian University of Science and Technology, Trondheim, Norway; ^8^Department of Computer Science, NTNU Norwegian University of Science and Technology, Trondheim, Norway; ^9^Department of Pharmacy, Health and Nutritional Sciences, University of Calabria, Rende, Italy; ^10^Department of Public Health and Nursing, NTNU Norwegian University of Science and Technology, Trondheim, Norway; ^11^Clinic of Laboratory Medicine, St. Olavs Hospital, Trondheim, Norway

**Keywords:** microRNA, non-coding RNA, breast milk, miRNA, ncRNA, extracellular vesicles, exosomes, RNA sequencing

## Abstract

Breast milk is the primary source of nutrition and hydration for the newborn infant but also plays an important role in the child’s first immune defense. Additionally, several breast milk factors have been implicated in immune-related health outcomes later in life, including immunoglobulins, cytokines, chemokines, growth factors and, more recently, non-coding RNA (ncRNA) species. In this systematic review, we provide a comprehensive summary of the current literature on endogenous ncRNAs found in human breast milk. Thirty (30) relevant studies were identified and, whilst the majority studies focused on microRNAs (miRNAs), there is evidence that breast milk contains high quantities of RNA which also include long-coding RNAs, circular RNAs, as well as other short RNAs and fragmented tRNA and rRNAs. Among studies investigating miRNAs, miR-148a-3p, miR-30a/d-5p, miR-22-3p, miR-146b-5p, miR-200a/c-3p, and the 5p end of the let-7 miRNAs were commonly reported among the top 10 miRNAs in the cell, lipid, and skim milk fractions of breast milk. Methodological difference and small sample sizes limit the possibility of conclusively identifying which maternal and infant characteristics affect the miRNA profile. The highly expressed miRNAs were generally reported to be similar across lactational stage, milk fraction, maternal and infant characteristics, or infant growth and health. All the same, individual studies identify potential differences in miRNA expression levels which should be confirmed by future studies. Stability, uptake, and physiological functions of miRNAs were also considered in several studies. Breast milk miRNAs are relatively resistant to a range of harsh conditions and uptake experiments suggest that extracellular vesicles containing miRNAs and circular RNAs can be taken up by intestinal epithelial cells. Although the evidence regarding the functional effect of breast milk miRNAs is limited, the predicted functions range from metabolic and biosynthetic processes to signaling pathways, cellular adhesion, communication, growth, and differentiation. Finally, this systematic review highlights some of the methodological challenges and knowledge gaps which can help direct future research in this field. In particular, it is important to further investigate the bioavailability of miRNAs in different milk fractions, and to characterize other ncRNAs which are largely unstudied.

**Systematic Review Registration:**

PROSPERO https://www.crd.york.ac.uk/prospero/display_record.php?RecordID=138989, identifier CRD42020138989.

## Introduction

Breast milk is the primary source of nutrition and hydration for the newborn infant and plays an important role in the child’s first immune defense (1–3). Several factors in the milk have potent immunological effects (4), such as maternal immune cells, secretory IgA, lysozymes and lactoferrin (1–3). These factors are transferred directly *via* breast milk from the mother to the infant, providing support to the naïve immune system. Indeed, breastfed infants have a lower rate of respiratory and gastrointestinal infections compared to formula-fed infants (3). Breast milk, however, also plays an important role in developing the infant’s own immune system (5–7) and protective effects from breastfeeding have been implicated in several immune-related health outcomes later in life (8, 9). This suggests that breastfeeding has immunological consequences beyond the breastfeeding period. Whilst investigations into the long-term effects of breast milk have conventionally focused on immunoglobulins, cytokines, chemokines and growth factors, breast milk also contains other components which may influence the developing immune system. For example, breast milk harbors a vast array of “non-coding” RNA species which could act as an alternate route contributing to the immune programming in infants, these molecules are far less explored than the more conventionally known breast milk components mentioned above.

It has been estimated that less than 2% of the transcripts from the human DNA actually code for proteins (10), hence the vast majority of these are so called non-coding RNA (ncRNA) sequences. In fact, it has been proposed that these ncRNAs contribute more to the biological complexity of eukaryotes, through sophisticated control of gene expression, than the actual protein coding genes themselves (11). The most well-known ncRNAs are transfer RNA (tRNA) and ribosomal RNA (rRNA). These RNA molecules play an integral role in the link between transcribed messenger RNA (mRNA) and mRNA translation to protein. The advent of high-throughput sequencing technologies, advancements in bioinformatics and biochemical approaches, have made it possible to identify and ascribe an increasing number of other ncRNA molecules to regulatory cellular processes, including regulation of chromatin structure, DNA transcription, RNA processing and stability and translation (12). A newcomer that has received a lot of attention in the past 15 years – both as biomarkers and as genes regulating normal and cancer development – is microRNA (miRNA).

The miRNAs are very short RNA molecules (20–24 nucleotides long) that can regulate protein expression post mRNA transcription (13, 14), primarily by destabilizing mRNA and inhibiting protein translation. Compared to other body fluids, breast milk is exceptionally rich in RNA (15); and many of the miRNAs found in breast milk are seemingly involved in modulating immunological pathways (16). Moreover, xeno-miRNA exhibiting maternal-infant immune cross-talk has also been found (17).

Breast milk miRNAs seem to remain stable in harsh environments (14, 18, 19) and recent research conducted on mice suggests that the concentration of extracellular vesicles (EVs) naturally found in milk whilst suckling is sufficient to result in accumulation of EVs in the tissues of piglets and mouse pups. Interestingly, these EVs may could subsequently be detected in a wide range of different organs, such as the heart, spleen, lungs, and brain (20). Hypothetically, breast milk miRNA could hence exert direct effects on immune regulation in the infant, for example by inhibiting the expression of key transcription factors in immune cell polarization (21, 22) or epigenetic modifications in immune cell linages (23, 24). Other regulatory ncRNA are also present in breast milk, such as long non-coding RNA (lncRNA), short interfering RNA (siRNA), piwi-interacting RNA (piRNA), circular RNA (circRNA) and fragmented tRNAs. Each of these ncRNA types have been found to have housekeeping or regulatory functions (25), with lncRNAs and circRNAs being the most widely studied (26, 27). The lncRNAs are RNA molecules of at least 200 nt commonly arising from splicing of two or more exons from genomic regions in proximity to protein-coding genes, including antisense and intronic sequences (27). The regulatory effect of lncRNAs has been attributed different modes of action from stabilization, maintenance and remodeling of chromatin loops to the binding of miRNAs, transcription factors, catalytic proteins or other chromatin-modification complexes (27). Post-transcriptional regulation through competitive binding of miRNAs is also found with circRNAs, and lncRNA and circRNAs have been described as miRNA sponges and competitive endogenous RNA (ceRNA) (26, 27). Circular RNAs (circRNAs) are generated by an unusual alternative splicing termed back-splicing, in which the 3′-end of an exon ligates to the 5′-end of its own exon, or to an upstream exon, to form a closed circular structure (28). They are ubiquitous in mammals and have been found to be functionally active both as miRNA sponges and through various circRNA-protein interactions (26, 29). Whilst functional consequence of lncRNAs and circRNAs in breast milk is incompletely understood, and their presence less studied than miRNAs, they may represent additional mechanisms by which breast milk can influence infant development.

This systematic review aimed to provide a comprehensive summary of the endogenous ncRNAs found in human breast milk, their stability, and potential functions, focusing on milk from healthy mothers. Further, we also aim to summarize current evidence for maternal and infant characteristics affecting the abundance of ncRNAs in the human breast milk of lactating mothers and associations between these ncRNAs and child health. Finally, we seek to provide guidance for future research within this field.

## Methods

### Protocol and Registration

This systematic review is registered in PROSPERO under ID CRD42020138989. The systematic review was initially submitted to PROSPERO in June 2019 and re-submitted after minor revisions following review from the PROSPERO editorial team in December 2019. The record was formally registered 7^th^ April 2020.

### Information Sources and Search Strategy

A comprehensive search was conducted by a medical research librarian (SAP) in the following electronic bibliographic databases: MEDLINE, Embase, The Cochrane Library (Cochrane Database of Systematic Reviews, Cochrane Central Register of Controlled Trials (CENTRAL), Cochrane Methodology Register), and Web of Science (Science and Social Science Citation Index). Briefly, the search strategy involved the two main concepts “non-coding RNA” and “breast milk”. Each concept was supplemented with an exhaustive list of synonyms and abbreviations using (the Boolean operator OR), before combining them (using the Boolean operator AND). For the concept “non-coding RNA”, we employed terms associated with known ncRNA including, but not limited to, miRNA, lncRNA, circRNA, siRNA, piRNA, rRNA and tRNA. The concept “breast milk” was expanded using terms such as human milk, colostrum, mother’s milk, breastfeeding, and lactation. The complete search strategies applied in the different databases are available in the **Supplementary Material**. All records identified in the search were imported into an EndNote library and duplicates were removed. The reference lists of eligible studies and relevant review articles were also screened to identify potentially relevant studies missing in the searched databases.

### Eligibility Criteria

All observational studies or clinical trials published in English were eligible for inclusion in that they reported analysis of human ncRNA in human milk, regardless of the laboratory method used, health care setting, or the maternal-infant characteristics and clinical health outcome investigated. Studies analyzing ncRNA in pathological lactation were excluded. *In silico* analyses were only included in the main presentation of results if the original study could not be included.

### Study Selection

Two researchers (LT and MRS) independently screened all the titles and abstracts of the unique records in the search results. Discrepancies were discussed between these two reviewers. Full texts of potentially eligible studies were retrieved and independently assessed for eligibility by the same two reviewers. When the eligibility of a particular study was unclear, this was discussed with the other review team members.

### Data Extraction

A standardized, pre-piloted spreadsheet was used to extract data from the included studies. Extracted information included: study setting; study population, participant demographics and baseline characteristics (incl. stage of lactation, gestational age at birth); breast milk collection methods (incl. time of day, fore/hindmilk collection, duration and temperature of storage); laboratory analysis methodology; child or maternal health outcomes (as appropriate); suggested target predictions and mechanisms of action. Data extraction was completed independently by four authors (LT, EA, LJ, and MRS) for articles which employed both quantitative-real-time-PCR (herein referred to as qPCR) and RNA sequencing (RNA-seq)-based methods of quantification. Two authors extracted the data independently for the remaining articles, with LT and LJ reviewing the studies using qPCR methods and EA and MRS reviewing those employing RNA-seq. Discrepancies were resolved through discussion in pairs and with a third author when necessary.

### Synthesis of Results

The findings of the included studies are summarized in a narrative synthesis, evaluating ncRNA abundance in human milk and their associations with maternal and infant characteristics and health outcomes. A meta-analysis was prohibited by the low number of studies reviewing any particular maternal or infant characteristic or child health outcome and the considerable heterogeneity in the study design and methods.

### Risk of Bias and Study Quality Assessment

The quality of the included studies was assessed using a checklist incorporating relevant questions from both NICE checklist for studies reporting correlations and associations (30) and the checklist suggested by Han et al. (31). In brief, we considered the clarity of the research questions and aims, the clarity of the description of the methods and results, and the risk of bias in the laboratory and statistical methods. Assessments were performed for each study by two reviewers (LT and MRS) and disagreements between the reviewers were resolved through discussion with the rest of the review team. The full version of the quality checklist can be found in the **Supplementary Material**.

## Results

### Study Selection and Characteristics

**Figure 1:**
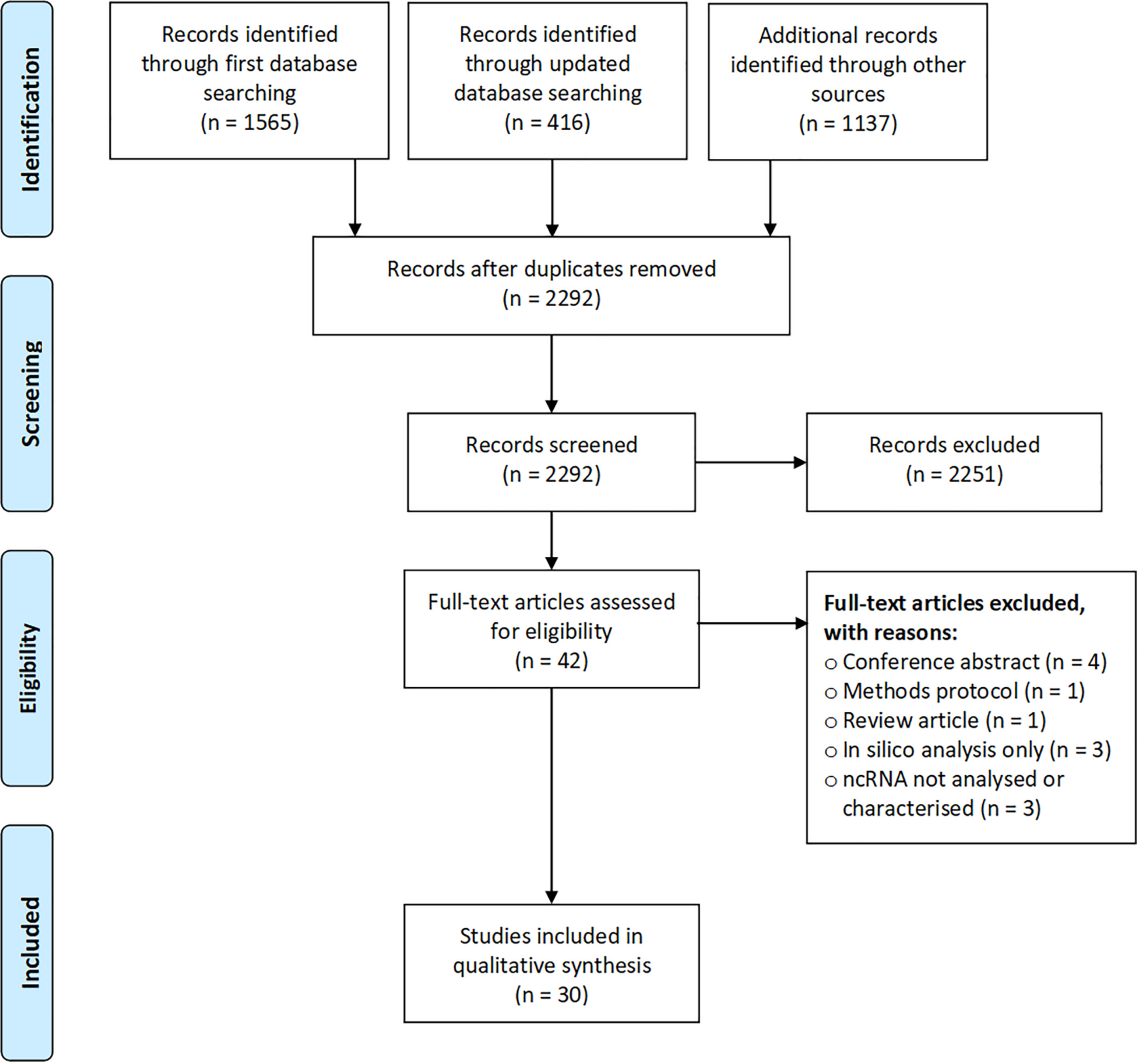
Flow diagram showing stages of study selection.

This review includes 30 studies describing ncRNA in human breast milk from lactating mothers (14, 15, 18, 19, 32–57). Our initial search conducted in March 2019 returned 1565 entries, of which 20 were ultimately included in this review (**Figure 1**). Examination of the reference lists of included articles and an update of the literature search in September 2020 returned a further seven articles (15, 36, 42, 47, 49, 51, 53), and a further three studies were published and integrated into our summary as we were finalising this manuscript (54–56).

The included studies were highly varied in their aims, design, and method of quantification (**Table 1**), as well as in their bioinformatic and statistical methods as described in later sections. Broadly speaking, the aims of the studies fell into one or more of the following categories: (a) investigation of ncRNA stability or uptake; (b) description of the general profile of human breast milk ncRNA; (c) identification of factors which may influence breast milk ncRNAs by assessing associations between maternal, gestational, or infant characteristics; and (d) examination of associations between ncRNAs and child health. In addition, some studies sought to determine the influence of methodological choices, such as sample storage, milk fraction analysed and RNA isolation protocol. With the exception of two studies which investigated lncRNAs (41) and circRNAs (54), the primary objective of all other studies revolved around miRNAs. As such, this review has a specific focus on human milk miRNAs with a brief description of other ncRNAs.

**Table 1:** Overview of the methods and aims.

Author (country)	Study aims	Milk volume to EV/RNA isolation	RNA amount/conc to ncRNA quantification	Number of women/samples	Lactation time	BM sample collection	BM fraction	EV isolation method	RNA isolation method	ncRNA profiling method
**Alsaweed et al.** (32) **(Australia)**	To standardize isolation of miRNA and total RNA from skim milk, lipid and cell fraction of human milk using eight commercially available kits	100-350 μL (unclear used volume for each kit) to RNA isolation	600 ng	29/49	3-158 w	Electric pump	Cell, lipid, and skim milk	NA	Eight RNA/miRNA filter column kits^a^	qPCR
**Alsaweed et al.^a^** (33) **(Australia)**	To characterize the cellular miRNA profile of human milk collected before and after feeding using next generation sequencing	Unclear (possibly 5mL)	500 ng/μL	16/20 (RNA-seq), 32 (qPCR)	4-8 w	Electric pump	Cell	NA	miRNeasy mini Kit	RNA-seq & qPCR
**Alsaweed et al.** (34) **(Australia)**	To profile 681 mature miRNAs in HM cells and lipid and compare them with maternal peripheral blood mononuclear cells (PBMCs), plasma, bovine and soy infant formulae using TaqMan OpenArray	Unclear (diluted 1:1 with PBS)	> 50 ng	10/10	4-8 w	Electric pump	Cell and lipid	NA	miRCURY RNA Isolation-Biofluids Kit (lipid). miRNeasy mini kit (cells)	qPCR
**Alsaweed et al.c** (35) **(Australia)**	To profile known and novel miRNAs in cell and lipid fraction collected at two, four and six months using deep sequencing	Unclear (5 mL diluted 1:1)	NR	10/30 (cell), 5/15 (lipid)	2, 4 & 6 mo.	Electric pump	Cell and Lipid	NA	miRCURY RNA Isolation-Biofluids Kit (lipid). miRNeasy mini kit (cells)	RNA-seq
**Bozack et al.** (36) **(USA)**	To investigate associations between the EV-miRNAs profile in breast milk and stressful events experienced over the mother’s lifetime or during pregnancy	Unclear (aliquots from freezer)	34 ng/μL	80/74	6.1 +/- 5.9 w	Manual pump	EVs from skim milk	exoEasy Maxi Kit	miRNeasy serum/plasma kit	qPCR
**Carney et al.** (37) **(USA)**	To evaluate the difference in miRNA profile in term and preterm breast milk	50 μL (lipid) and 200 μL (skim milk) (to EV isolation)	NR	44/67	48 h & 3-4 w	Manual or electric pump	EVs from Lipid and skim milk	Norgen circulating and Exosomal RNA Purification Kit	Norgen circulating and Exosomal RNA Purification Kit	RNA-seq
**Floris et al.** (38) **(France)**	To evaluate a miRNA assay to minimize the amounts of raw milk obtained from mothers of preterm infants, and to investigate miRNA expression within two months of lactation and over the course of 24 hours	50, 100 and 300 μL (whole milk) unclear vol for lipid and skim milk. (To RNA isolation)	4 ng	22/84	2 mo.	NR	Whole, lipid and skim milk	NA	Organic phase separation	qPCR
**Golan-Gerstl et al.** (39) **(Israel)**	To determine the miRNA profile in skim and lipid fraction of human, goat, and bovine milk as well as infant formulas using next generation sequencing and quantitative real-time PCR. Further, the biological effects of the milk miRNAs were evaluated on normal and transformed intestinal cells	NR	100 ng (RNA-seq)/400 ng (qPCR)	13/13 for qPCR; unclear for RNA-seq	1 mo.	NR	Lipid and EVs from skim milk	Centrifugation, 5 and 0.45µm filtration and ExoQuick	Organic phase separation and miRNeasy mini kit and the RNeasy MinElute Cleanup kit	RNA-seq & qPCR
**Khan et al.** (40) **(USA)**	To determine the miRNA profile in preterm milk exosomes and to investigate the uptake ability in intestinal epithelia cells after gastric/pancreatic *in vitro* digestion	20 mL (to EV isolation)	NR	20/40	6 days - 5 w	Electric pump	EVs from Skim milk	Centrifugation, 0.45 µm filtration and ExoQuick	SeraMir Exosome RNA Amplification Kit	RNA-seq
**Karlsson et al.** (41) **(USA)**	To determine if extracellular vesicles isolated from human milk contains developmentally related long non-coding RNAs using a custom real time PCR array	1 mL (to EV-RNA isolation)	8.5 ng	30/30	2 mo.	Manual pump	EVs from Skim milk	Centrifugation, 0.8 µm filtration and ExoEasy Maxi Kit	ExoRNeasy Serum/Plasma Maxi Kit	qPCR
**Kosaka et al.** (14) **(Japan)**	To determine the miRNA profile in human milk with in the first 6 months of lactation, and to further determine the miRNA stability after RNase and freeze-thawing treatment	Unclear (sample vol 50-100 mL)	70 ng (microarray)/NR (qPCR)	8/Unclear	4 days - 11 mo.	NR	EVs from Skim milk	Anti-CD63 sorting	mirVana miRNA isolation kit	Microarray and qPCR
**Kupsco et al.** (56) **(Faroe Islands)**	To determine expression of human milk EV-miRNAs in a large population, characterize miRNA clusters and potential biological functions of these miRNAs using gene ontology; and examine associations of miRNAs with maternal body mass index (BMI), smoking, parity, and collection date.	1-2 mL	30 ng	364/364	2 - 74 days	Hand expression or by pump	EVs from skim milk	ExoEasy Maxi kit	miRNeasy Serum/Plasma Maxi kit	HTG EdgeSeq tecnhology
**Leiferman et al.** (42) **(USA)**	To lay the methodological groundwork for studies of miRNA in exosomes from small sample volumes of human milk in large cohorts of women, and assess exosome and miRNA content in infant formulas	1 mL (EV isolation)	NR	5/3 (both RNA-seq & qPCR)	2–10 mo	NR	EVs from skim milk	Ultra-centrifuagtion	miRNeasy micro kit	RNA-seq & qPCR
**Liao et al.** (18) **(USA)**	To investigate the effects of *in vitro* digestion on milk exosomes, explore the uptake in an intestinal epithelial model, and further elucidate miRNA sensitivity to digestion at early-, mid-, and late lactation by next generation sequencing	20 mL (to EV isolation, unclear to ExoQuick)	NR	12/24	1.5-8 mo.	Breast pump	EVs from Skim milk	Centrifugation, 0.45 µm filtration and ExoQuick	SeraMir Exosome RNA amplification kit and TRIzol	RNA-seq
**Munch et al.** (43) **(USA)**	To compare miRNA profile in human milk from two different cohorts: One cohort where the RNA was isolated before and following a short-term treatment with recombinant human growth hormone. A second cohort which was used to evaluate 12 novel miRNAs in a large validation set that included lactating women consuming enriched diets	NR	NR	22/39 for qPCR 3/6 for RNA-seq	6-12 w	Breast pump	Lipid and EVs from Whole milk	ExoQuick	Tri-Reagent RNA isolation and mirVANA isolation	RNA-seq & qPCR
**Na et al.** (44) **(China)**	To compare the expression of immune-related miRNAs in human, black goats, and cattle milk	NR	NR	3/3	< 7 d	NR	NR	NA	RNAiso for Small RNA kit	qPCR
**Perri et al.** (45) **(Italy)**	To evaluate the expression of four immune-related miRNAs in colostrum and mature milk using qPCR	200 μL (to RNA isolation)	NR	33/33	NR	Electric pump	Whole milk	NA	miRNeasy Serum/Plasma Kit	qPCR
**Qin et al.** (57) **(USA)**	To determine if cancer-related miRNAs are present in breast milk fractions and serum from lactating women.^b^	NR	NR	6/6	NR	Pumped	Lipids, skim milk and cells	NA	miRNeasy micro kit	qPCR
**Rubio et al.** (46) **(Spain)**	To compare the miRNAs, isomiRs and small RNA profiles in plasma and milk	Unclear	500 ng	10/10	48-72 hrs	Electric pump	Skim milk	NA	miRNeasy Serum/Plasma kit	RNA-seq
**Shah et al.** (55)	To test if maternal obesity and infant anthropometrics during the first six months of life is associated with specific EV-related miRNAs in HM	2 mL	NR	60/108	1 & 3 months	Electric pump	EVs from skim milk	ExoQuick	SeraMir Exosome RNA Isolation kit	qPCR
**Shiff et al.** (47) **(Israel)**	To investigate if highly expressed miRNAs differ between milk from mothers from term and pre-term infants, during stage of lactation and fractions of breast milk. Additionally, the biological effects were tested *in vitro*	NR	400 ng	38/76 (15 pre-term and 23 term)	0-48 h and 30 d	Manual or electric pump	Lipid and skim milk	NA^c^	Trizol and chloroform	qPCR
**Simpson et al.** (48) **(Norway)**	First, to determine the miRNA profile in human milk, second, to examine if this profile is influenced by maternal probiotic supplementation and third, to assess if any changes in the miRNA profile are associated with the development of Atopic dermatitis in the offspring	1.5 mL (to ExoQuick)	NR	54/54	3 mo.	NR	EVs from Skim milk	Centrifugation and ExoQuick	miRNeasy kit	RNA-seq
**Smyczynska et al.** (49) **(Poland)**	To evaluate the total and exosome-bound miRNA content in human milk comparing unpasteurized milk to two different pasteurization preservation methods.	5 mL (to miRCURY)	NR	3/3	50 d	NR	Whole milk and EVs	miRCURY Exosome Cell/Urine/CSF Kit	miRNeasy Serum/Plasma Advanced Kit	RNA-seq
**van Herwijnen et al.** (50) **(Netherlands)**	To compare the miRNA profile in extracellular vesicle between human milk and other mammals	NR	8 ng	4/1 (pooled)	3-9 mo.	NR	EVs from Skim milk	Ultracentrifugation and sucrose density gradient	miRNeasy Micro kit	RNA-seq
**Weber et al.** (15) **(Sweden)**	To examine the spectrum of miRNAs in 12 body fluids, including breast milk and colostrum	300 μL (skim milk to RNA isolation)	NR	5/1 (mature milk) 1/1 (colostrum)	NR	NR	Skim milk	NA	miRNeasy kit	Human miScript Assay
**Wu et al.** (51) **(China)**	To investigate the miRNA expression profile in human colostrum compared to mature milk	NR	NR	18/18 (18 qPCR; 4 microarray)	1-7 d and 14 d	By hand	Skim milk	NA	EasyPure miRNA Kit	Microarray and qPCR
**Xi et al.** (52) **(China)**	To investigate the expression of three miRNAs in colostrum and mature milk. Further, the effects of maternal and infant characteristics on the three miRNAs expression were also evaluated	250 μL (to RNA isolation)	NR	86/119	48 h – 3 mo.	NR	Skim milk	NA	QIAzol and miRNeasy Mini Kit	qPCR
**Zamanillo et al.** (53) **(Spain)**	To investigate how maternal weight affect the miRNAs expression and their association with milk levels of leptin and adiponectin, as well as their impact on infant BMI at two years of age	100 μL (to RNA isolation)	2.5 ng/μL	59/unclear^d^	1, 2 and 3 mo.	Spontaneous aspiration from opposite breast during feeding or manual pump	Whole milk	NA	mirVana microRNA Isolation Kit	qPCR
**Zhou et al.** (19) **(China)**	To investigate the exosome miRNA expression in human milk using deep sequencing to profile immune-related miRNAs. Further, resistance and stability of miRNAs under different harsh conditions were evaluated	1 mL (to ExoQuick)	NR	4/4	60 d	Manual pump	EVs from skim milk	Centrifugation, 0.45 μm filtration and ExoQuick	TRIzol-LS	RNA-seq & qPCR
**Zhou et al.** (54) **(China)**	To evaluate differences in EV-related circRNAs between preterm and term colostrum samples and their role in regulation of intestinal development.	50 mL	NR	18/6 pooled samples (2-4 mothers per pool)	0-30 days	NR	EVs from skim milk	Ultracentrifugation	TRIzol Reagent and a pure tissue kit (Tiagen)	qPCR

a miRNeasy micro Kit, Qiagen., mirVana microRNA Isolation Kit, Ambion., RNAzol-RT Reagent, Molecular Res. Center., miRNeasy mini Kit, Qiagen., TRIzol-LS Reagent, Invitrogen., miRCURY RNA Isolation-Cell & Plant Kit, Exiqon., miRCURY RNA Isolation-Biofluids Kit, Exiqon., mirPremier microRNA Isolation Kit, Sigma–Aldrich.

b Despite aiming to investigate the presence of “cancer-related miRNAs” the women included were healthy lactating mothers without personal history of breast cancer and thus the analyses represent physiological expression of the miRNAs.

c EV isolation with ExoQuick for subsequent uptake experiments (RNA isolated from lipid and skim milk fractions).

d 48-55 samples reported per miRNA per timepoint.

In terms of methods, the studies can also be grouped according to which milk fraction was investigated or which method of quantification was employed. Twenty studies reported ncRNA quantification results from a single breast milk fraction, including skim milk with EV isolation (14, 18, 19, 36, 40–42, 48, 50, 54–56), skim milk without EV isolation (15, 46, 51, 52), cells (33), lipids (43) and whole milk (45, 53) (**Table 1**). Two studies analyzed the lipid fraction along with skim milk (37) or EVs (47) without directly comparing the results between the fractions, while seven of the remaining studies provide results from the direct comparison of ncRNAs in different milk fractions (32, 34, 35, 38, 39, 49, 57). Milk fraction was not reported in one study (44). Similarly, the majority of studies investigated ncRNAs in breast milk using a single method of quantification (k = 23 studies): ten studies employed targeted qPCR analyses only (32, 36, 38, 44, 45, 47, 52, 53, 55, 57), four used only array technologies (15, 34, 41, 54), and nine studies used RNA sequencing technology only (18, 35, 37, 40, 46, 48–50, 56). The remaining studies used a combination of qPCR with either RNA sequencing (19, 33, 39, 42, 43) or microarray (14, 51). The number of participating mothers and included samples ranged from 3 to 364, with some studies collecting more than one sample per mother (**Table 1**).

Among the studies which employed qPCR, a range of chemistries have been used to analyse miRNAs (**Table 2**). Two studies used custom TaqMan arrays (33, 43), three used available TaqMan OpenArrays (34, 36) or Human miScript Assay (15), one study used an array based on SYBR Green chemistry to analyse 87 lncRNAs (41) and one used the Arraystar Human circRNA Array to investigate circRNAs (54). The remaining studies analysed between two and thirteen specific miRNAs using TaqMan, SYBR Green or EvaGreen chemistries (14, 19, 32, 38, 39, 42, 44, 45, 47, 51–53, 55, 57). The use of endogenous and exogenous controls varied between the studies, with a large proportion of studies lacking either or both of these controls (**Table 2**). Whilst 17 of the 21 included qPCR-based studies described the data normalisation, the method and level of detail on the procedure varied. All the studies which employed RNA-seq used an Illumina platform for sequencing, yet the choice of library preparation kits differed between studies, as did the bioinformatic pipelines for processing, aligning and normalisation of reads (**Table 3**). None of these studies reported using spiked-in synthetic miRNA in the sequencing protocol.

**Table 2:** Methods of quantification in studies using qPCR and miRNA investigated.

Author	PCR chemistry	Endogenous control	Exogenous control	Normalization	microRNA analyzed
**Alsaweed et al.** (32)	TaqMan primer probe	RNU48	NR	Relative expression to endogenous control	miR-30a-5p, miR-148a-3p
**Alsaweed et al.** (33)	Custom TaqMan Small RNA	NR	NR	Unclear	let-7f-5p, miR-181a-5p, miR-148a-3p, miR-22-3p, miR-182-5p, and novel miRNAs referred to as: novel-mir-7-5p, novel-mir-299-5p, novel-mir-367-3p and novel-mir-39-5p
**Alsaweed et al.** (34)	TaqMan miRNA OpenArray panel system	RNU48, RNU44 and RNU6	ath-miR159a	Yes however, no details provided.	681 mature miRNAs
**Bozack et al.** (36) **(USA)**	TaqMan OpenArray 384	miRNeasy Serum/ Plasma Spike in (RNU6)^1^	ath-miR159a ^2^	Global mean method	752 known miRNAs
**Floris et al.** (38)	TaqMan primer probe	NR	Cel-lin4-5p	Geometric mean of endogenous miRNAs	miR-16-5p, let7g-5p, let-7a-5p, let-7d-5p, miR-146b-5p and hsa-miR-21-5p
**Golan-Gerstl et al.** (39)	SYBR Green	RNU6	NR	Relative expression to endogenous control	miRNA-148^a^, miRNA-206, miRNA-375, miRNAs-320, miRNA-146^a^ and miRNA-146b
**Karlsson et al.** (41)	SYBR Green lncRNA array	NR	NR	NR	87 lncRNAs
**Kosaka et al.** (14)	TaqMan primer probe	NR	Cel-miR-39	Relative expression to exogenous control	miR-181a, miR-17, miR-155, and mir-92
**Leiferman et al.** (42)	miScript SYBR Green	NR	miSpike	NR	miR-30d-5p, miR-125a-5p, and miR-423-5p
**Munch et al.** (43)	Custom TaqMan Small RNA	RNU6	NR	Relative expression to endogenous control.	Novel miRNAs referred to as: novel-miR-102, novel-miR-79, novel-miR-54, novel-miR-114, novel-miR-37, novel-miR-111, novel-miR-67, novel-miR-109, novel-miR-27, novel-miR-120, novel-miR-123, novel-miR-44, novel-miR-112, novel-miR-113, novel-miR-118.2, novel-miR-135, novel-miR-138, novel-miR-62, novel-miR-68, novel-miR-26, novel-miR-126. Sequences are available in **Supplementary File** associated with Munch et al.
**Na et al.** (44)	SYBR Green	NR	NR	NR	mir-146, mir-150, mir-155, mir-181a and mir-223
**Perri et al.** (45)	TaqMan primer probe	NR	ath-miR159a	Absolute quantification using exogenous control	hsa-miR-21, hsa-miR-181a, hsa-miR-150 and hsa-miR-223
**Qin et al.** (57)	miScript SYBR Green PCR kit	Snord95	NR	Relative expression to andogenous control	miR-10a-5p, miR-16, miR-21, miR-100, miR-140, miR-145, miR-155, miR-181, miR-199, miR-205, miR-212
**Shah et al.** (55)	TaqMan Assay	NR	NR	Geometric mean of all samples	miR-148a, miR-30b, miR-29a, miR-29b, miR-let-7a and miR-32
**Shiff et al.** (47)	Perfecta SYBR Green SuperMix	RNU6	NR	Relative expression to endogenous control	hsa-miR-320a, hsa-miR-148a-3p and hsa-miR-146a-5p. Additionally, miR-375 is reported in the results but not described in the methods section.
**Weber et al.** (15)	Human miScript Assay	NR	NR	Global mean	714 different human miRNA species (600 detected in at least 1 of 12 body fluids; 419 and 386 detected in breast milk and colostrum, respectively).
**Wu et al.** (51)	SGExcel Fast SYBR Mixture	NR	Cel-miR-39	Relative expression to exogenous control	hsa-miR-623, hsa-miR-885-5p, hsa-miR-429, hsa-miR-511-3p, hsa-miR-29c-3p, hsa-miR-183-5p and hsa-miR-30b-5p
**Xi et al.** (52)	TaqMan primer probe	NR	Cel-miR-39	Relative expression to exogenous control	miRNA-30b, let-7a and miRNA-378
**Zamanillo et al.** (53)	TaqMan primer probe	RNU6 and hsa-miR-539-5p (negative control)	NR	Using fixed small RNA input to cDNA. Relative expression to endogenous control	miR-148a, miR-181a, miR-222, miR-103, miR-30a, miR-27a, miR-27b, miR-200b, miR-let7a, miR-17, miR-let7b, miR-let7c and miR-146b
**Zhou et al.** (19)	EvaGreen	NR	ath-miR-159a-3p, cel-lin-4-5p and cel-miR-2-3p	Relative expression using Cel-lin-4-5p and cel-miR-2-3p as references.	let-7a-2-5p & -3-5p, let-7f-1-5p & -2-5p, miR-29a-3p, miR-30b-5p, miR-141-3p, miR-146b-5p, miR-148a-3p, miR-182-5p, miR-200a-3p and miR-378-3p
**Zhou et al.** (54)	Arraystar Human circRNA Array v2, and SYBR	NR	NR	Quantile normalization (limma package)	6756 circRNAs detected (unclear how many included in array)

1. The kit also included RNU44 and RNU48, but these were not used in the normalization of samples.

2. ath-miR159a was included in the kit, but not used.

**Table 3:** Methods of quantification in studies using RNA sequencing.

Author	RNA quality check	Library kit	Seq platform	Quality control and processing of raw reads	Alignment database	Normalization method	Open data
**Alsaweed** (33)	Bioanalyzer 2100, RNA 6000 Nano Chip kit	Solexa small RNAs protocol	Illumina HiSeq 2000 (read length not reported)	Low reads or contaminate reads such as 5´ primers, no insert tags, oversized insertion, low quality reads, poly A reads, etc. were removed. The tool used was not described.	Clean reads annotated by BLAST into categories using: • Rfam and GenBank to remove other ncRNA (no version given) • Degraded fragments of mRNA removed after alignment to exons and introns • Remaining reads mapped to miRBase 21.0	normalized expression = actual miRNA count/total count of clean reads x 1,000,000.	GSE71098
**Alsaweed** (35)	Bioanalyzer 2100, RNA 6000 Nano Chip kit	Solexa small RNAs protocol	Illumina HiSeq 2000 (read length not reported)	Low reads or contaminate reads such as 5´ primers, oversized insertion, and reads shorter than 18 nt were removed. Quality was also checked in individual samples. The tool used was not described	• SOAP software • Human genome (no version given) • Reads aligned to Rfam and GenBank to remove other ncRNA (no version given) • Degraded fragments of mRNA removed after alignment to exons and introns • Remaining reads mapped to miRBase 21.0 using BLAST	normalized expression = actual miRNA count/total count of clean reads x 1,000,000.	GSE75726
**Carney et al.** (37)	Agilent 2100 Bioanalyzer (Chip kit not specified)	NEXTflex Small RNA-Seq Kit v3 (Bioo Scientific).	Illumina HiSeq 2500 (read length not reported)	FASTX Toolkit Module was used to clip, trim and filter reads to max. read length of 30 bp	• Human genome (hg38) using Bowtie 2 algorithm • Total miRNA counts within each sample quantified with miRBase mature miRNAs v21.0	normalized across samples using a trimmed mean of M-values (TMM) method.	NR
**Golan-Gerstl et al.** (39)	Agilent Bioanalyzer 2100 system (Chip kit not specified)	NEBNext R _ Multiplex Small RNA Library Prep	Illumina NextSeq 500 (read length not reported)	NR in article; refers to pipeline described in Leshkowitz et al. (58): Perl scripts and the crossmatch tool were used to screen and clip the adaptor sequence and then to update tag frequencies.	NR in article; refers to pipeline described in Leshkowitz et al. (58): Clean reads annotated by BLAST and mapped to miRBase 18.	Reported as percentage of total miRNA reads	NR
**Kahn et al.** (40)	Agilent Bioanalyzer 2100 (Chip kit not specified)	TruSeq Small RNA sample preparation kit	Illumina HiSeq 2500 (paired end sequencing, read length not reported)	NR	NR	Described as counts per million, although unclear normalization method on review of **Supplementary Tables**.	NR
**Kupsco et al.** (56)	Implen NanoPhotometer and TapeStation 4200 total RNA ScreenTape chips	EdgeSeq miRNA Whole Transcriptome Assay (probe-based library preparation)	Illumina HiSeq 400 (read length not reported)	Quality requirements included ≥85% with a quality score greater than 30 (Q30 score) in each cluster, and percentage of clusters passing flter≥75%, and 180<CD<290.	NA	Median ratio normalization method in DESeq2 package	GSE146880
**Leiferman et al.** (42)	Fragment Analyzer Automated CE System	NEXTflex Small RNA-Seq Kit v3	Illumina Hiseq 2500 (read length not reported)	QC was done using FastQC. Adaptor sequences and reads containing ambiguous bases or having an average quality score of < 30 were removed using Cutadapt	Reads were aligned against miRBase version 22 using miRDeep2.	Reads were normalized by library size and multiplied by a factor of 10^6^, which corresponds to counts per million mapped miRNA reads.	PRJNA477819
**Liao et al.** (18)	Agilent 2100 Bioanalyze, Agilent small RNA kit	TruSeq Small RNA sample preparation kit	Illumina Hiseq 2500 (read length not reported)	The qrqc software was used to assess read quality and contaminants. Scythe and Sickle were used to remove adapter and low-quality bases.	• miRDeep2 program alignments to • miRbase v.20	Report normalization with described method; unclear on review of **Supplementary Tables**.	SRP081347
**Munch et al.** (43)	Unclear	NR	Illumina 1G Genome Analyzer, 36 nt sequencing reads	Reads described as trimmed and filtered using an in-house program	• miRbase release 16. *human genome (NCBI 36/UCSC hg18). • Vienna package to identify hairpins. Reads within hairpins were further filtered to remove miRBase release 18 miRNA species.	Quantile normalization of reads from known miRNA	NR
**Rubio et al.** (46)	Agilent RNA 6000 pico chip and Agilent 2100 Bioanalyzer	NEBNext Small RNA Library Prep Set	Illumina HiSeq 2000, single-end 50 bp	QC was conducted using FASTX-Toolkit and FastQ Screen. After adaptor removal, reads with the following features were removed: Reads < 18nt, Mean PHRED scores < 30, and Low complexity reads based on mean score of the read.	• miRBase v21 using miraligner to detect miRNAs and isomiRs • hs37d5 using bowtie and hotspots identified • Hotspots with >60% sequence sharing annotated to different RNA species using miRBase v20, refGene, wgRna, rmsk and tRNAs.	Normalization using DESeq 2 v1.10.1 – internal normalization where geometric mean is calculated for each gene across all samples (scaling factor method).	GSE107524
**Simpson et al.** (48)	Agilent Bioanalyzer 2100 using the Agilent RNA 6000 Nano Kit/Small RNA kit	ScriptMiner Small RNA-Seq Library Preparation Kit	Illumina HiSeq 2000, single-end 50 bp	Raw reads were processed with cutadapt. Low-quality bases < 20 were removed from reads before adapter removal and the final reads were required to have a length of > 17 bp.	• human genome with STAR v.2.4.0, requiring a perfect match alignment. • featureCounts v.1.4.0 was used to map miRNAs in miRBase v.20	Normalized to total reads matched to mature miRNA (count per million; CPM)	**Supplementary File**
**Smyczynska et al.** (49)	TapeStation 2200 using HS-RNA kit	QIAseq miRNA Library Kit	Single end Illumina NextSeq 500 sequencer, single-end 75 bp	FastQC, MultiQC and custom scripts were used for QC. Read adapters were trimmed and UMI sequences were extracted by UMI-tools v1.0.0. Maximum 2 bp mismatch in 19 bp-long adapter sequence was allowed. Reads with incomplete UMI sequence (< 12 nt) and shorter than 15 bp were excluded.	Reads were mapped to miRBase v22, prior miRNA records with identical sequences were collapsed to allow unique mapping using Bowtie2 v2.3.4.1. Reads not mapped to miRBase, were aligned to hg38 using the Burrows-Wheeler (BWA 0.7.17)	Read counts were transformed to transcripts per million (TPM). Presented as percentage of mature miRNA reads.	GSE142282
**van Herwijnen et al.** (50)	Agilent 2100 Bioanalyzer and Pico 6000 RNA chips	NebNext small RNA library prep kit	Illumina HiSeq 2000 (read length not reported)	Data quality was checked with FastQC, and reads were processed with cutadapt (v1.8) to remove low quality reads. Sequences with a length of > 15 bp after adapter trimming were retained.	• miRNA hairpin sequences using bowtie v1.1.1. • alignments to miRbase v.21	NR	GSE118409
**Zhou et al.** (19)	Agilent Bioanalyzer 2100 and the RNA 6000 Nano LabChip Kit	NR	Illumina Genome Analyzer II, single-end 36 bp reads	Do not describe method used. In brief, the raw reads were passed through a series of filters, such as length and sequence comparison	• miRBase 17.0	Presented as percentage of miRNA reads.	GSE32253

### Synthesis of Results

#### NcRNA and miRNA Profile

The majority of studies focused on miRNAs and, among those using RNA-seq, the proportion of cleaned reads which mapped to miRNAs range between 0.6% (50) to nearly 65% (33) (**Table S1, Supplementary Material**). Whilst most studies failed to provide details about the origins of the remaining reads, some reported significant proportions of rRNA and tRNA fragments (46, 48, 50), as well as some reporting smaller fractions of snoRNAs, snRNA and piRNAs (33, 35, 46). Due to their focus on miRNAs, other ncRNAs observed in these studies are primarily limited to small RNAs (~18 to 30 nt) depending on the width of the size selection step after library preparation. However, prior to library preparation, Bioanalyzer results indicate significant quantities of RNAs up to 500 nt and possibly 1000 nt (41, 43, 48). Detailed examination of the origins and function of longer ncRNAs is limited to two articles which have investigated lncRNAs (41) and circRNAs (54). Fifty-five of the 87 developmentally related miRNAs investigated in Karlsson et al. (41) were expressed in EVs from at least one of the 30 breast milk samples analyzed. Five of these lncRNAs were consistently expressed in 90-100% of the samples, including CRNDE, DANCR, GAS5, SRA1 and ZFAS1 which may be involved in metabolism, adipogenesis and immune cell regulation. More recently, Zhou et al. (54) expanded the exploration of human milk ncRNAs and identified 6756 circRNAs associated with milk EVs using a microarray based platform. The overall circRNA profile was not presented in this study, instead they identified differentially expressed miRNAs between term and preterm milk and investigated potential functions of the differentially expressed circRNAs as described below (54).

**Figure 2:**
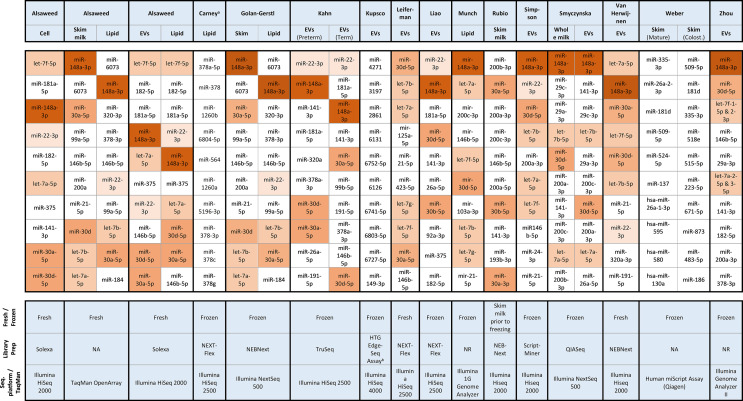
Top 10 mature miRNA from RNA-seq and TaqMan OpenArray studies. Colost., Colostrum; EV, Wxtracellular vesicles; NA, not applicable; ^a^Carney et al. (37) assessed lipid and skim fraction, however the top 10 miRNA could only be deduced for the lipid fraction in the **Supplementary Table** on differential expression between term and preterm milk which reports total reads per miRNA, ^b^Kupsco et al. (56) employed HTG EdgeSeq miRNA Whole Transcriptome Assay, a novel sequencing platform with probe-based library preparation. Bozack et al. (36) and Wu et al. (51) also used array-based technologies, however these articles do not supply information regarding the top 10 miRNAs.

Concerning the more widely studied miRNAs, the 10 most commonly occurring miRNAs in the 16 RNA-seq and array-based studies which report on the overall profile are summarized in **Figure 2**. These top 10 miRNAs represented between ~60 to 80% of miRNA reads in most sequencing studies (**Table S1, Supplementary Material**). In contrast, two studies from the same laboratory reported that the top 15 miRNAs accounted for only 10 – 11% of mature miRNA reads (18, 40). On further inspection, these proportions appear to be calculated based on logarithmically transformed normalized reads. The raw read counts presented for one of the articles indicate that the top 10 miRNAs actually account for 63.8% of miRNA reads (18). The majority of studies found that miR-148a-3p and miRNAs from the let-7 and miR-30 families were among the top 10 most highly expressed miRNAs, and around half of the studies reported miR-22-3p, miR-146b-5p and miR-200a/c-3p among the top 10 (**Figure 2**). From the miR-30 family, miR-30a-5p and miR-30d-5p were the most commonly expressed with 13 of 16 studies reporting either or both of these miRNAs observed among the top 10. Similarly, members of let-7 family were identified as highly expressed in 10 studies. Interestingly, these highly expressed miRNAs appear to be observed in fresh and frozen milk, and across different milk fractions, library preparation methods and sequencing platforms. Notable exceptions to this fairly consistent finding was the study by Rubio et al. (46) which did not observe miR-148a-3p among the top 10 and the studies by Carney et al. (37), Weber et al. (15) and, most recently, Kupsco et al. (56) which observed markedly different miRNA profiles.

#### Novel miRNA Candidates

In addition to detecting known miRNAs, sequencing methodologies can also be used to identify novel miRNA candidates as seen in three of the included studies (33, 35, 43). Broadly speaking, the methodological approaches for identifying novel miRNA candidates were similar: sequences that were mapped to the human genome, but could not be matched to miRBase or other known RNA species, were analyzed further as possible novel miRNA candidates through the identification of potential precursor miRNA structures (hairpins) using the softwares Mireap (33), mirdeep (35) and Vienna (43). Munch et al. (43) specified that the 100 bp flanking sequence was included when searching for hairpin structures, and it was implied that the identification of hairpins was conducted before removal of miRNAs annotated in miRBase v18.0 (given the initial alignment was conducted in v16.0), snoRNAs, scaRNAs, repeats identified by Repeat-Masker, and reads with high GC content (>90%). On the other hand, in two studies by Alsaweed and coauthors (33, 35), reads mapping to ncRNAs and mRNA fragments were removed prior to hairpin structure identification. These two studies also evaluated base bias on the first position and the nucleotide length on each position in the miRNA candidates, however it is unclear how this information was used in the later analyses. Munch et al. (43) reported only novel miRNA candidates which were present in at least two sequencing runs. In contrast, Alsaweed and coauthors provide information on all novel miRNA candidates identified with at least one read in at least one sample, although they defined novel miRNA candidates as “high-confidence” if at least 20 reads were identified in at least three (35) or four (33) samples.

An overview of the top 10 novel miRNA candidates reported in each of these papers can be found in **Table S2** (**Supplementary Material**). In the two Alsaweed et al. papers (33, 35), 1999 and 5167 novel miRNA candidates were reported across 20 and 45 samples, respectively. However, closer inspection of these sequences reveals that these are not all distinct miRNA candidates as some differ by only one nucleotide at the 3’ or 5’ end of the sequence, suggesting they come from the same genomic location. Failure to align these sequences with each other has overestimated the number of novel miRNA candidates in these studies but has also underestimated the read counts for some novel miRNA candidates since the reads are spread across several entries. Regardless of their uniqueness, the candidate miRNAs accounted for only 31,233 and 916,090 reads in these two articles, representing 0. 01% and 0.3% of the 174,186,532 and 281,181,648 reads matched to known miRNAs in miRBase v21.0. This was also the case for the 21 novel miRNA candidates identified in Munch et al. (43) who report a total of 4759 novel miRNA candidate reads in 6 samples, compared to 31,102,927 reads perfectly matched to miRBase v16.0. As such, the reproducibility and biological significance of most of these novel miRNA candidates is uncertain. The more highly expressed novel miRNA candidates were validated with qPCR in two of the studies (33, 43), which supports the hypothesis that they are truly present rather than potential sequencing errors.

#### NcRNA Stability

To exert physiological effects in the offspring, breast milk miRNAs need to reach their destination, or target tissue, intact. Seven (14, 18, 19, 38, 40, 45, 49) of the included papers performed miRNA stability experiments. One of the papers studying uptake also included a stability experiment after pasteurization (39), however only in cow and goat milk, and since this review focuses on human milk, the stability comparison of this study was omitted from our result section. The experiments for evaluating miRNA stability in human milk involved subjecting milk samples or milk vesicles to various treatments, such as freezing (38) or freeze-thaw cycles (14, 19), heat incubation (19) or pasteurization (45, 49), as well as conditions aiming to mimic gastric digestion including acid (14, 18, 40), RNase (14, 19) and pancreatin treatment (18, 40) (see **Table 4** for a comprehensive overview).

**Table 4:** Stability assessment of ncRNA in human milk.

Author, year	Sample	Stability assessment	Result
**Floris et al.** (38)	Fresh whole milk	Storage in -80°C for 24h and 1 month, respectively.	No significant difference was found between fresh and stored milk, at least for miR-16, miR-21, let-7a, let-7g and let-7d.
**Kahn et al.** (40)	Frozen whole milk or EVs (Unclear if previously isolated EVs or whole milk used in these experiments)	Mimic of gastric digestion by HCl treatment (pH 4.0) combined with pepsin and 20 min incubation at 37°C, followed by pH normalization (pH 7.0) and pancreatin treatment for 30 min in 37°C.	*In vitro* digestion does not have any pronounced effects on milk vesicle miRNA expression.
**Kosaka et al.** (14)	Whole milk/skim milk (unclear)	60 min RNase treatment Freeze-thaw cycles Incubation in low pH solution (pH 1.0)	Treatment with RNase had no or only slight effect on human breast milk miRNAs. By contrast, the profile of total RNA from that milk and exogenously added synthetic miRNAs were degraded by the same treatment (data not shown in paper). The endogenous miRNAs were also stable after freeze thaw treatment and low pH incubation.
**Liao et al.** (18)	Fresh whole milk (EVs isolated afterwards for further analysis)	Mimic of gastric digestion by HCl treatment (pH 4.0) combined with pepsin and 20 min incubation at 37°C, followed by pH normalization (pH 7.0) and pancreatin treatment for 30 min in 37°C	The top 288 miRNAs in digested milk vesicles showed no significant difference after digestion.
**Perri et al.** (45)	Whole milk	Pasteurization of whole breast milk at 62.5°C for 30 minutes, compared to instantly frozen breast milk (-80°C).	Expression levels hsa-miR-21, hsa-miR-181a, hsa-miR-150 and hsa-miR-223, in skim milk, did not change significantly due to the treatment.
**Smyczynska et al.** (49)	Fresh whole milk and EVs	Holder pasteurization (HoP) of whole breast milk at 62.5˚C for 30 minutes. High pressure processing (HPP) in 450 MPa for 15 min.	A greater preservation of miRNAs seen in the HPP samples.
**Zhou et al.** (19)	Whole (fresh)? milk with added exogenous synthetic miRNA	Incubation in 26oC for 0.5 1, 2, 4, 8 and 24 hours. Repeated freeze-thaw cycles at -20oC. 60 min RNase treatment at 37oC. 10 min incubation at 100oC.	In contrast to the complete degradation of synthesized exogenous miRNAs, endogenous miRNAs were partially resistant to prolonged room temperature incubation, multiple freeze-thaw cycles, RNase treatment and incubation at 100°C. However, these treatments also resulted in a ~40 to 90% reduction in miRNA expression depending on the treatment and miRNA.

The effect of sample storage was reported in four studies. Floris et al. described no substantial difference in the abundance of miR-16, miR-21, let-7a, let-7g or let-7d when comparing fresh milk samples to aliquots stored at -80 degrees for 24 hours or 1 month (38). Similarly, Kosaka et al. (14) reported relative stability of miR-21 and miR-181a for up to three freeze-thaw cycles, although both of these studies included only two samples from two women in their assessments. In contrast, the findings of Zhou et al. (19) suggest that miRNA expression may be 20 to 40% lower than their original values after three freeze-thaw cycles, and as much as 60% lower after six freeze-thaw cycles. However, it is important to note that this study clearly demonstrates that endogenous miRNAs are substantially more stable than the exogenous *Arabidopsis thaliana* miRNA used as a control which was undetectable after six freeze-thaw cycles. Munch et al. (43) also present Bioanalyzer results from the lipid fraction of fresh and frozen milk demonstrating that fresh samples have bands corresponding to 18S and 28S rRNA which are absent in samples processed after freezing. Whether or not freezing influence the overall miRNA/ncRNA profile could however not be further determined as only frozen samples were analyzed using RNA-seq and qPCR (43).

The influence of pasteurization on breast milk miRNAs has been assessed in two studies (38, 49). Overall, these studies suggest that pasteurization may affect specific, but not all, miRNAs and the method of pasteurization may influence the effect on miRNA abundance. Whilst Perri et al. (45) reported no change in the content of selected immune-related mRNAs in whole milk after standard Holder pasteurization (HoP), RNA-seq analyses recently reported by Smyczynska et al. (49) suggest that there was substantial degradation of miRNAs in whole milk following HoP. On the other hand, reads of miRNA-length were still found in EVs after HoP without further investigating which miRNAs were differentially expressed in this milk fraction. Smyczynska et al. (49) study also investigated the influence of high-pressure pasteurization (HPP), a process which appeared to have less of an effect on human milk miRNAs but still resulted in altered relative abundance of several miRNAs in whole milk and EVs. In particular, the miRNAs miR-29c-3p, miR-29a-3p and miR-374a-5p were observed to be significantly reduced in both whole milk and EVs following HPP, and a number of other miRNAs were altered in either whole milk or the EV fraction.

#### Uptake Experiments and Functional Predictions

In order to further support proposed physiological effects of breast milk miRNAs, experiments to clarify whether milk vesicles are taken up by cells were conducted in four studies, from two research groups. Additionally, the uptake of vesicles and potential function of circRNAs was investigated by Zhou et al. (54). Each of these studies investigated if milk vesicles, most commonly referred to as exosomes, were internalized in cell cultures of different kinds. We have opted to use the term EVs instead of exosomes and comment further on the distinction between them in the Discussion (§4.6). Liao et al. (18) used human intestinal epithelial crypt-like cells (HIEC) and detected milk EVs *via* confocal microscopy by fluorescent dyes. The cells were incubated with the milk vesicles for 30 min and 2 hours. Based on their experiments, the authors suggest that the EVs are taken up by the cells continuously during this time period. Kahn et al. (40) used the same HIEC cell line in a much similar fashion; these studies originate from the same research group. The HIEC cells were grown to 60% of confluence and incubated with serum-free medium for 2 h at 37°C, then treated with the milk EVs and subsequently fixed. Confocal microscopy showed internalization of milk EVs in the cell cultures and nuclear localization was also observed. In these two studies, milk vesicles from both term and pre-term infants were included and all results support internalization by the HIEC cells, also after subjecting EVs to an *in vitro* gastric digestion protocol involving treatment with hydrochloric acid, pepsin and pancreatin (**Table 4**).

Golan-Gerst et al. (39) and Shiff et al. (47) performed additional downstream analyses to explore functional effects from the EV uptake using different cell lines and incubation procedures, demonstrating some reproducibility in their findings. Golan-Gerst et al. (39) used a human colonic cell line of fetal origin (CRL-1831), a leukemia cell line (K562) and a colorectal cancer cell line (LIM1215). The cell cultures were incubated with fluorescent labeled milk vesicles for 2 h and analyzed by microscopy. After the incubation, all cell cultures were found positive for vesicle uptake. The authors could also show a biological effect of the vesicle-incubations: vesicle-treated cells upregulated miRNA-148a expression as compared to control with a subsequent down regulation of DNA-methyltransferase 1 (DNTM1), a target gene of miR-148a. In a later study from the same research group, Shiff et al. (47) studied the effects of a 24 h milk vesicle incubation on colonic cancer cells (LS123), colon epithelial cells (CCD 841) and hepatocarcinoma cells (HUH7). In line with their previous study (39), they observed an increase of miRNA-148a in CCD 841 and subsequent downregulation of DNMT1. Additionally, the experiments showed an upregulation of miRNA-320 in all three cell lines, with a simultaneous decrease in expression of the target enzyme fatty acid synthase. These experiments used changes in miR-320 expression as a proxy for exosome uptake, without actually confirming the uptake by immunofluorescence (47). Also using a colonic cell line (FHC), Zhou et al. investigated EV uptake in their efforts to understand the potential physiological role of circRNAs in breast milk (54). In their experiments, Zhou et al. (54) demonstrated that milk EVs promoted proliferation and migration of intestinal epithelial cells, and that vascular endothelial growth factor (VEGF) appears to play a central role in this process. Whilst these functional assays did not directly assess the role of circRNAs, *in silico* target gene analyses of the circRNAs identified in the milk samples, together with their predicted miRNA interactions, indicated that the VEGF signaling pathway was the most highly enriched pathway (54).

Another approach used to predict physiological functions of the breast milk miRNAs is performing *in silico* target and functional annotation analysis, which was included in 11 studies (see **Table 5** for an overview of the method and results). The most common prediction tool used was TargetScan (33, 35, 43, 48, 49, 51, 56), with the other papers using range of tools including miRanda (33, 35, 43), RNAhybrid (33, 35, 56), Tarbase (36, 50), microT-CDS (37, 56), miRDB and miRNA.com (51), miRror 2 Suite (53), QIAGEN’s Ingenuity Pathway Analysis software (35), or circBase (54). Additionally, all papers presented Gene Ontology (GO) terms and Kyoto Encyclopedia of Genes and Genomes (KEGG) pathways in their functional analysis. The functional annotation tools used included DIANA mirPath version 3 online software (36, 37, 49), DAVID (43, 48, 54), Funrich version 3 (50), Cytoscape 3.7.2 (51), and GO and GlueGo CluePedia Cytoscape plugin (53). In summary, between 19 target genes and 50 300 target transcript variants were identified. Even though the number of targets differ between the studies, the functional analysis produced similar results; the most common GO terms and KEGG pathways are related to metabolic processes and immunological function (**Table 5**).

**Table 5:** miRNA gene target analysis and functional prediction.

Author	Dataset for gene target prediction	Gene target prediction tool	Functional analysis tool	Gene targets (n)	Main results
**Alsaweed et al.** (33)	Top10 highest expressed known and novel miRNA. Top 23 known miRNA	Targetscan, RNAhybrid and miRanda. QIAGEN’s IPA	GO and KEGG	17 586 and 13 066unique transcript variant targets for known and novel miRNA, respectively (TargetScan). Top 23 miRNA: 8 925 unique gene targets (IPA)	MiRNA targets were involved in immune responses, development, growth, metabolic processes, reproduction, and exert enzyme regulatory activity; target genes seem to be mainly involved in pathways related to metabolic networks, such as glycerophospholipid metabolism, porphyrin metabolism, and nitrogen metabolism.
**Alsaweed et al.** (35)	Top 20 highest expressed known and novel miRNA	TargetScan, RNAhybrid and miRanda	GO and KEGG	~ 50 300 potential target transcript variants	Target genes were mainly involved in programmed cell death, cell-to cell communication, cell adhesion, peptide transport, nervous and immune system development, and metabolic processes.
**Bozack et al.** (36)	Eight and 17 miRNAs related to LSCR and NLE score, respectively.	Tarbase v.7.0	DIANA miRPath 3	19 and 30, respectively	Top enriched pathways included fatty acid biosynthesis and fatty acid metabolism, Hippo signaling pathway and steroid biosynthesis
**Carney et al.** (37)	15 miRNAs altered in lipid fraction between pre *vs* term milk, 12 miRNAs altered in skim milk between pre *vs* term milk and 9 miRNAs altered in lipid and skim milk between pre *vs* term milk	microT-CDS	DIANA mirPath 3	4 401, 2 767 and 2 614 mRNA targets, resp*	Taking together the results from both lipid and skim milk fraction, nine miRNAs were differently expressed in preterm milk *vs* term milk. According to functional analysis, those nine miRNAs seem to be involved in metabolic pathways; the top GO process terms included, cellular nitrogen metabolism and biosynthetic process. The most enriched KEGG pathways were glycosphingolipid biosynthesis and lysine degradation.
**Kupsco et al.** (56)	miRNAs from the three highest expressing clusters	DIANA microT-CDS and TargetScan; Tarbase v7.0	DIANA miRPath version 3	Top 15 miRNA (Cluster 1) were reported to have between 21 and 969 predicted targets each. Functional analysis based on all miRNAs in the top three clusters. Total number of targets not reported.	KEGG pathways common to the top three expression clusters were: Fatty acid biosynthesis, adherens junction, focal adhesion, ECM-receptor interaction, signaling pathways regulating pluripotency of stem cells, platelet activation, hippo signaling pathway, endocytosis, Rap1 signaling pathway, thyroid hormone signaling pathway, and PI3K-Akt signaling pathway. GO terms for the miRNA in Cluster 1 included: organelle, cellular nitrogen compound metabolic process, biosynthetic process, symbiosis encompassing mutualism through parasitism, ion binding, small molecule metabolic process, and neurotrophin TRK receptor signaling pathway
**Munch et el (** 43 **).**	All and top 10 of known miRNA and 12 novel miRNAs	TargetScan and miRanda	DAVID	9 075, 2 691 and 3 554, unique potential gene targets, respectively*	The most enriched GO terms for the top 10 known miRNAs were regulation of transcription, metabolic processes, and biosynthetic processes. Insulin signaling and Axon guidance were the most enriched KEGG pathways. With a less stringent Benjamini multiple testing correction, they also found GO terms and KEGG pathways related to the immune system. The 12 validated novel miRNAs showed similar terms as the known miRNAs, as well as the following terms; plasma membrane, regulation of T cell receptor signaling pathway, ion binding and calcium ion binding.
**Simpson et al.** (48)	Top 20 highest expressed miRNA and the differentially expressed after probiotic treatment and associated with atopy	TargetScan v 7.0	DAVID 6.7	3 498, 495 and 2 269 unique potential gene targets, respectively*	The target analysis showed involvement in the positive and negative regulation of metabolic processes involving nitrogen compounds, RNA, DNA and macromolecules and the positive regulation of transcription and gene expression, embryonic development, angiogenesis, catabolic processes and cell migration and localization.
**Smyczynska et al.** (49)	miRNAs which accounted for 90% of total miRNA reads in unprocessed samples were used for target prediction. Further, targets of 8 miRNAs which suffered the biggest depletion during HPP was also included in the analysis.	TargetScan	DIANA mirPath 3	NR	Top enriched pathways in both whole and exosome samples included ECM-receptor interaction, prion disease, fatty acid biosynthesis and focal adhesion.
**van Herwijnen et al.** (50)	NR	Tarbase v 7.0	Funrich 3	24	Seven GO terms were identified and seems to be involved in the following biological processes, regulation of gene expression epigenetic, cell communication, signaling transduction, cell growth and/or maintenance, transport, energy pathway and metabolism.
**Wu et al.** (51)	10 colostrum specific miRNAs	MiRDB, TargetScan and microRNA.org	Cytoscape 3.7.2	260	MiRNA targets were related to the molecular functions; protein serine/threonine kinase activity, chromatin binding, ubiquitin-protein transferase activity, and nucleotide binding. Cellular component; was mainly in the nucleus, cytoplasm, and cytosol. Biological process; was involved in signal transduction, positive regulation of GTPase activity, protein phosphorylation, intracellular signal transduction. Top enriched KEGG pathways included MAPK, PI3K-Akt, endocytosis, focal adhesion and Ras signaling.
**Zamanillo et al.** (53)	Target prediction was done on all selected miRNA (n=26)	miRror 2.0 Suite	GO and GlueGo + CluePedia Cytoscape plugin	487	The miRNA targets were related to epithelial cell development, neuromuscular process, Wnt signaling pathway and calcium modulating pathway, regulation of macroautophagy, central nervous system neuron differentiation, lipid particle organization, Golgi vesicle transport, cranial nerve development, and cell morphogenesis involved in differentiation.
**Zhou et al.** (54)	Unclear	CircBase used to predict targets.	DAVID (version not reported)	NR	GO enrichment and signal pathway analysis indicated that circRNAs were involved in a range biological processes, molecular function, and pathways, and associated with different cellular components. KEGG pathway analysis indicated that the most significant pathway was the VEGF signaling pathway. Involvement in VEGF signaling was further investigated in *in vitro* experiments.

*Respectively for each dataset uploaded for target analysis.

DAVID, Database for Annotation, Visualization, and Integrated Discovery, Funrich, Functional Enrichment analysis tool, GO, Gene Ontology, IPA, Ingenuity Pathway Analysis; KEGG, Kyoto Encyclopedia of Genes and Genomes.

#### Factors Associated With ncRNA Profile

Twenty-one of the thirty included studies formally compared the abundance of specific miRNAs and or the general miRNA profiles between two or more groups. The most common comparison was between lactational stages, although the stages assessed varied between studies (14, 18, 35, 37, 38, 44, 45, 47, 51–53, 55, 56). Other factors assessed for their association to miRNAs included milk sample processing (35, 38, 43, 49), milk fraction (32, 35, 38, 39, 43, 47, 49, 57), RNA extraction method (32), fore- or hind milk collection (33, 37), diurnal variation (38), preterm delivery (37, 40, 47, 54), infant sex (36, 52), maternal characteristics (36, 43, 48, 52, 53, 55, 56), and infant health and development (48, 53, 55).

##### Lactational Factors, Gestational Age, miRNAs and circRNAs

Since lactational stage and gestational age are known to influence other bioactive components of breast milk, it is feasible that miRNA concentrations and their relative abundances also change over time and with gestational duration. The influence of lactational stage was assessed for specific miRNAs (14, 35, 38, 45, 47, 52, 53, 55) and for the miRNA profile more generally (35, 37, 51, 56). Whilst miR-21 was consistently reported as being stable across a range of lactational stages in multiple studies (35, 38, 45), other miRNAs were less widely studied. Perri et al. (45) found similar quantities of miR-21, miR-181a, miR-150 and miR-223 between colostrum and mature milk samples. Similarly, Floris et al. (38) found that miR-21, miR-16 and mi-146b displayed quite stable levels between one to two months post-delivery. They also reported that let-7a, let-7d and let-7g had slightly greater variability over this period without providing details of how these miRNAs differed. Analyzing highly expressed miRNAs in samples collected at 1 and 3 months postpartum, Shah et al. (55) reported a slight decrease in miR-148a, an increase in miR-30b and no apparent change in let-7a in the later lactational stages. In contrast, Shiff et al. (47) reported no apparent change in miR-148 from zero to one month postpartum in skim milk, and a non-statistically significant increase in the lipid fraction of milk from mothers of full-term infants. The same study suggested that miR-320 abundance may decrease from zero to one month after a full-term delivery in both the skim and lipid fractions (47). Alsaweed et al. (35) found that most of their top 20 miRNAs in sequencing analyses were relatively stable in both the cellular and lipid fraction of samples collected at two, four and six months post-delivery. These highly expressed miRNAs included miR-21-5p, miR-181a-5p, miR-146b-5p, let-7a-5p which are among the miRNA described as stable in other studies. Looking beyond six months post-delivery, Kosaka et al. (14) reported higher relative expression of miR-181a, miR-17, miR-155 and miR-92 in samples collected between zero to six months post-delivery compared to those collected after six months. Additionally, their findings suggest that samples from the same mother are highly correlated over time, and less strongly correlated with samples from other women at the corresponding time period.

Despite this apparent stability in highly expressed and common miRNAs, Carney et al. (37) and Wu et al. (51) report overall differences in colostrum and mature milk miRNA profile. Using partial least squares discriminant analysis (PLS-DA), Carney found partial separation of the general profile in their three sample types: colostrum and mature milk samples from mothers of term infants and mature milk samples from mothers of preterm infants. However, there is insufficient information in these studies to identify which miRNAs may be driving the differences seen between colostrum and mature milk (37, 51).

The influence of gestational age on individual miRNAs was investigated through the comparison of term and preterm human milk in three studies (37, 40, 47), and by considering correlations between gestational age as a continuous variable and miRNAs in two studies (37, 52). Whilst three (37, 40, 47) of these four studies report differences which may be associated with gestational age, the findings are difficult to compare between the studies. On the whole, Carney et al. (37) and Kahn et al. (40) found that most miRNAs expressed in term milk could also be detected in preterm milk and *vice versa*, and that miRNAs found exclusively in preterm- or term milk had low abundance (40). When comparing term and preterm milk, Carney et al. (37) described 113 and 12 differentially expressed miRNAs in the lipid and skim fractions of mature milk, respectively. The corresponding statistical comparisons are not presented in Kahn et al. (40) who focused on the effect of digestion on very *versus* extremely preterm milk. Using qPCR, Shiff et al. (47) failed to find clear differences in miR-146, miR-148, miR-320 or miR-375 in mature milk samples, which is consistent with the results from Carney et al. for these miRNAs. On the other hand, Shiff et al. observed higher miR-148 and lower miRNA-320 levels in skim and lipid fraction from colostrum samples from women who had delivered preterm. Lastly, the one study investigating circRNAs in breast milk also considered the effect of gestational age on these ncRNAs (54) and reported 66 up- and 42 downregulated circRNAs in preterm versus term milk. Although they focused on circRNA with at least a two-fold change between the preterm and term samples, there is some inconsistencies between the tables presented in this study and there are no other studies in the field to compare the reproducibility of their findings.

Other lactational factors which may affect the miRNA profile are the collection of fore- or hindmilk and diurnal variations. The quantity of miRNA extracted from breast milk appears to be similar in fore- and hindmilk (33, 37). Whereas Alsaweed et al. (33) reported that 339 miRNAs were identified as fore- or hindmilk specific and a further 33 miRNAs found in both fore- and hindmilk were differentially expressed, none of these miRNAs were abundant. Specifically, the ones found uniquely in one sample type each accounted for between 1 and 35 reads in less than five samples, and those reported as present before and after feeding, but differentially expressed, each accounted for less than 0.5% of total miRNA reads (33). Only one study has investigated diurnal variations in breast milk miRNAs (38), and found that miR-146b, let-7d and let-7g were relatively stable throughout the day, miR-16 had a relatively higher expression in the evening, while miR-21 and let-7a were more variable without a clear diurnal pattern.

##### Milk Fraction and miRNAs

The choice of breast milk fraction may also influence the concentrations of specific miRNAs and the overall miRNA profile. Alsaweed and co-workers have published most comprehensively on the influence of breast milk fraction, reporting the cellular fraction as the milk fraction containing the highest concentration of total RNA and miRNA, followed by the lipid fraction and lastly the skim milk fraction (32, 34, 35). We noted that these studies reported total RNA and miRNA quantities in the cellular fraction as nano- or micrograms per million cells without apparent standardization to a whole milk volume, which makes comparison with the concentrations from other milk fractions difficult. The relative abundance of miRNAs has been compared between lipid and cellular fraction (34, 35), between lipid and skim milk fractions (38, 39), and between all three of these milk fractions (57). Additionally, one study investigating lipid and skim milk also compared these fractions to the miRNA concentrations recovered from whole milk (38). Whilst each of these studies found that some miRNAs were unique or differentially expressed in specific milk fractions, these findings were inconsistent across the studies. Furthermore, many of the top 10 miRNAs appear to be identified as highly expressed in all milk fractions (**Figure 2**).

##### Maternal Characteristics and miRNA Profile

The influence of several maternal characteristics on the miRNA profile of breast milk has been investigated, including associations with maternal age (36, 52), parity (56), ethnicity (36), weight or body mass index (BMI) (36, 52, 53, 55, 56), diet (43), probiotic supplementation (48), smoking (36, 56), as well as gestational diabetes mellitus and hypertension (52) and stressful events during the mother’s life and pregnancy (36). The findings of these studies are summarized below, but it should be noted that none of the studies have investigated the same maternal factors against the same set of miRNAs, and it is therefore difficult to merge the results across the studies.

Whilst Xi et al. (52) found no conclusive evidence of correlation between maternal age and let-7a, miR-30b or miR-378 in colostrum, they did observe that each of these miRNAs were negatively correlated with pre-pregnancy weight and BMI and, to a lesser degree, with post-pregnancy weight and BMI. They also described an association between these miRNAs and both gestational diabetes mellitus and gestational hypertensive disorders, which disappeared after adjusting for pre-pregnancy BMI. Associations between maternal BMI and breast milk miRNAs were also investigated by Zamanillo et al. (53) and Shah et al. (55). While the findings of Xi et al. (52) suggest that higher BMI was associated with lower let-7a in colostrum, none of these studies found a conclusive correlation between BMI and let-7a in mature milk (52, 53, 55), the only miRNA analyzed in all three studies. Shah et al. (55) and Zamanillo et al. (53) both included analyses of miR-148a but found partially conflicting results. Shah et al. otherwise observed reduced relative amounts of miR-29a, miR-29b and miR-30b in overweight and obese women at 1 month postpartum, and Zamanillo et al. (53) observed a higher average abundance of miR-30a, miR-103 and miR-222 in milk from overweight and obese mothers at one- and three-months post-partum. At the same time, these associations were attenuated or reversed at two months post-partum (53).

Using RNA-seq on samples collected during a randomized controlled trial, Simpson et al. (48) failed to find a convincing association between maternal probiotic supplementation and the relative abundance of individual miRNAs at three months gestation (48). Specifically, they observed an upregulation of let-7d-3p and downregulation of miR-574-3p, miR-340-5p and miR-218-5p, although the false discovery rate (FDR) was unacceptably high. Munch et al. (43) also analyzed samples from mothers assigned to different dietary interventions, however formal comparisons of miRNA abundance between groups were only conducted for the novel miRNA candidates, which were present in very low relative abundance as described above (§3.2.2), and the general miRNA profile was only assessed using RNA-seq in three women. Bozack et al. (36) considered the psychosocial influences on breast milk miRNA and found that lifetime stress and negative life events in mothers appeared to be associated with the abundance of specific miRNA, although the multiple comparisons are not taken into consideration in their statistical analyses.

##### MiRNA Profile and Infant Sex and Health

In terms of infant characteristics and outcomes, only few studies have investigated associations between the breast milk miRNA profile and infant sex (37, 52), growth or development (53, 55) or health (48). Xi et al. (52) reported that let-7a, miR-30b and miR-378 levels were higher in milk of mothers who gave birth to female babies compared to male babies, with this observation being statistically significant in the latter two miRNAs. Whilst Carney et al. (37) also examined associations between infant sex and a subgroup of miRNAs, including several versions of miR-378, none of these were statistically significant and there is insufficient information about the direction of the associations to allow a comparison to the findings of Xi et al. (52). Regarding infant growth, Shah et al. (55) reported that infant weight and body composition over the first six months of life was not consistently associated with miR-148a, miR-29a, miR-29b, miR-30b, let-7a or miR-32 in one- or three-month milk samples (55). At two years of age, infant BMI was negatively correlated with a range of milk miRNAs in the study by Zamanillo et al. (53), including let-7a, let-7b, let-7c, miR-17, miR-27a, miR-27b, miR-103, miR-30a, miR-146, miR-148a, miR-181a, miR-200b and miR-222. With the exception of miR-27a, miR-27b and miR-30a, all of these correlations were statistically significant or borderline non-significant. In the case of miR-17, miR-103, miR-146b, miR-181a, and miR-222, there was also some indications that the correlation between these miRNAs and infant BMI at two years was stronger when the mother herself had a BMI under 25 kg/m2. In terms of infant health, the only study identified was Simpson et al. (48) which analyzed associations between infant eczema and milk miRNAs, in addition to the effect of maternal probiotic supplementation described above. Whilst this study found 13 miRNAs had a p-value below 0.05, these findings were interpreted cautiously since the FDR for all comparisons was greater than 0.10 and there was uncertain clinical significance after considering the predicted function of these miRNA.

### Assessment of Study Quality

Overall, there was an adequate level of detail in the information on study design, research questions, aims and inclusion of participants (**Table S3**). A major limitation for most studies was however the small number of women and samples included. Only two of the studies reported a sample size calculation, although there are currently no widely accepted sample size calculation methods for RNA-seq analyses which can be easily applied *a priori* without utilising data from a pilot study (59). Furthermore, details about the sample collection and storage were often incomplete. Few articles described the timing of sample collection with respect to time of day, or whether fore- or hindmilk was collected, and around one third of studies (11 of 30) did not report how the samples were collected (e.g., pump or manuals expression).

The description of the laboratory analyses was generally sufficient, although some key details were either unclear or unreported in many studies such that replication of the methods would be difficult. For example, the volume of milk used in the analysis was unclear or unreported in 14 of the studies (**Table 1**), and only seven studies (37–39, 45, 47, 48, 52) reported what volume was used in the elution step of RNA extraction. We note here that differences in the initial volume of milk or elution volume should theoretically not influence the relative abundance of miRNAs but introduces methodological variability. Five studies reported methods which involved different protocols for different groups of samples which were later compared. Specifically, different starting volumes of milk were used in the assessment of RNA kits (32), different kits were used for RNA extraction in different milk fractions (34, 35) and samples appear to stem from distinct cohorts with slightly different collection procedures (37, 43). Whilst all studies analysed biological replicates, technical replicates were employed in seven of the 21 studies with qPCR-based analyses and none of the RNA-seq analyses.

Details regarding the processing and statistical analysis of the data was also highly variable (**Tables S3, S4** and **S5, Supplementary Material**). Particularly among the RNA-seq and high-throughput array studies, only 8 of 20 studies used widely accepted statistical packages for analysis of differential expression in RNA-seq data, such as limma (60) used by four studies (34, 43, 48, 54), DESeq2 (61) used by two studies (49, 56) and edgeR (62) used by another two studies (18, 51). These packages account for multiple hypothesis testing through estimation of a False Discovery Rate (FDR). Another two studies (33, 35) used the less common DEGSeq package (63) which can also calculate a FDR, although it was unclear if this was used in the presentation of the results. The remaining RNA-seq studies either did not conduct any formal comparisons of the sequenced milk miRNAs (15, 19, 39, 42, 46, 50) or employed simpler methods such as Students t-test (18, 40) or Mann Whitney (37) without adjusting for the multiple comparison, or regression models not implemented in one of the aforementioned packages (36) (**Table S5**). We also identified a number of uncertainties around the normalisation for both PCR and RNA-seq experiments. For studies using PCR quantification methods, the use of a standardised concentration of RNA prior to cDNA synthesis was reported in many, but not all studies (**Table 1**), as was normalisation to endogenous and exogenous controls (**Table 2**). Among the RNA-seq studies, the most common method of normalisation was to calculate reads per million for each miRNA, however in two studies it was unclear if this had been performed on a group or individual level (33, 35) and in the case of Kahn et al. (40) and Liao et al. (18) the reported read counts appear to have possibly undergone a logarithmic transformation after normalisation.

## Discussion

This systematic review aims to provide a comprehensive summary of the endogenous ncRNAs found in human breast milk focusing on milk from healthy mothers and include the results from 30 scientific peer-reviewed studies. Given most of the included studies specifically investigated miRNAs, with no or limited description of other ncRNA species, this review also focuses primarily on miRNAs in human milk. Even among studies investigating miRNAs, we found that the studies were heterogeneous with little overlap between their specific objectives. Consequently, no collective analysis is included in this review, apart from the synthesis of the top expressed miRNAs in human breast milk. This is a common feature for young and fast-growing research fields as the coherence in methods and other study features are continually developing. Furthermore, heterogeneity, lack of standardisation of sample collection and processing within and between studies is a known challenge in breast milk research (64–66). The studies included in this review were no exception and, like others before us, we identified a need for greater standardisation and clearer reporting of sample collection and processing methods which we discuss below (64, 66). Looking beyond the methodological limitations and challenges, we first discuss the current state of knowledge regarding the general miRNA profile in human milk, their stability and function and factors which affect them.

### Non-Coding RNAs in Human Breast Milk

Compared to other body fluids, human breast milk contains high quantities of RNA (15). At the same time, only a fraction of these RNAs have been investigated and characterized. Most human milk studies in this field have focused on miRNAs, and those using high-throughput technologies provide insight into the general miRNA profile and a glimpse of the small ncRNA profile. Other ncRNA species have not been thoroughly characterized in human milk, nor has there been any functional or stability experiments investigating RNAs such as tRNA and rRNA fragments, piRNAs, snoRNAs and snRNAs. As such, their role and function in human milk is unclear. On the other hand, lncRNAs and circRNAs have been specifically investigated in two human studies (41, 54). Whilst there also remains a need for further research into the profile and function of these ncRNAs, both studies report a wide range of potential functions for human milk lncRNAs and circRNAs. These RNAs species have also been described in bovine (67, 68) and porcine (69) milk samples, although it is unclear if the same lncRNAs and circRNAs are being observed across animal species.

MicroRNAs are more widely studied and the RNA-seq and array-based studies demonstrate a number of similarities among the highly expressed miRNAs. In particular, miR-148a-3p, miR-30a/d-5p, miR-22-3p, miR-146b-5p, miR-200a/c-3p, and the 5p end of the let-7 miRNAs were generally reported among the top 10 miRNAs in the cell, lipid and skim milk fractions and using different analysis protocols. Furthermore, no miRNAs were consistently found to be differentially expressed between these milk fractions. The significance of the overall similarity in the miRNA profile and lack of reproducible differences between breast milk fractions is unclear, although it may indicate that the miRNAs have a similar origin and function regardless of the milk fraction. Previous analyses have suggested that mammary epithelial cells are likely to be the primary origin of milk miRNAs in both the lipid and cellular fraction (34, 70). Mammary epithelial cells are the dominant cell type in human milk (71), and release of their miRNAs could possibly be the source of miRNAs in all fractions of milk, including EVs.

When considering the overall miRNA profile, the recent study by Kupsco et al. (56) warrants particular attention. This study is both the largest analysis of miRNA in human milk to date, but also the only study to have employed HTG EdgeSeq technology to investigate 2083 mature miRNAs. The overall miRNA profile reported in this study differs substantially form other recent sequencing analyses of both human and other mammalian milk. Given the stark difference in the reported miRNA profile, it would have been interesting to conduct confirmatory qPCR analyses assessing the relative abundance of the highly expressed miRNA in their study (e.g., miR-4721 and miR-3197) and miRNAs which are reported as highly expressed in other studies (e.g., miR-148a-3p, miR-30a-5p and let-7a-5p). It is probable that the difference is at least partially due to the probe-based library preparation method used with HTG EdgeSeq, which does not necessarily distinguish between the mature miRNAs targeted by the probes and other RNAs, including longer species, that may hybridize to the same probes. This potential for false positive off-target signals distinguishes the HTG EdgeSeq method from the more traditional library preparation using adapter ligation and reverse transcription, which instead are more prone to distorted signals from ligase biases.

As could be expected, the majority of miRNAs identified in breast milk through RNA-seq were known miRNAs. Among the studies which sought to identify novel miRNA candidates (33, 35, 43), these were found at very low relative abundances and often in few samples. Reliable identification of novel miRNA candidates would require larger quantities of data from a greater number of milk samples and systematic methods which consider typical features of miRNA. Based on a recent validation study, the falsely-positive novel miRNA candidates was reported to be 65% after *in silico* analyses and only around 18% could be confirmed on functional experiments (72). Therefore, *in silico* should be complemented with northern blot assays, functional assays seeking to demonstrate Drosha/Dicer dependence or Ago protein binding, and confirmation of conservation across species (73).

### Stability, Uptake and Function of Breast Milk ncRNAs

From experimental studies reviewed here, it may be concluded that breast milk miRNAs are reasonably stable, have the potential to be taken up by intestinal epithelial cells with functional consequences. Particularly when compared to exogenous miRNA controls, breast milk miRNAs appear to be relatively resistant to freezing or freeze-thaw cycles (14, 19, 38), heat incubation (19), pasteurization (45, 49), acid (14, 18, 40) and RNase treatment (14, 19), as well as pancreatin treatment (18, 40). Most studies however were small and examined vesicle enclosed miRNAs where the vesicle membrane may constitute an important protective factor, as compared to miRNAs that are free in solution or protein bound.

The stability evaluation is interesting from methodological, physiological, and clinical perspectives. Methodologically, we can ask if studies of frozen breast milk be compared to studies of fresh samples? In the studies evaluating fresh *versus* frozen breast milk (38) as well as freeze-thaw treatment (14) of samples, the results suggests that at least some of the highly expressed miRNA remain consistent. However, others have shown that the preparation of the samples with either RNA extraction or fractioning of the milk should preferably be done when the sample is still fresh, otherwise content from lysed cells may change the miRNA profile of the sample (74). Physiologically, the stability of miRNAs is a prerequisite for maintenance of biological function, and we can ask do miRNA survive the harsh condition of the infant’s digestive system? The four studies investigating this premise simulated digestion of different breast milk fractions and concluded that the treatments had no significant, or only slight, effects on the miRNA expression in the examined fractions (14, 18, 19, 40). It is worth remembering here that a minimum of 1,000 copies of miRNAs are likely required for a biological effect inside the cell (75), although some experimental evidence suggests that as few as 100 copies may be sufficient (76). Finally, it is of clinical interest to understand if freezing and or pasteurization affect the miRNA when mothers or donors provide expressed breast milk. Currently, when own mother’s milk is unavailable, or is in short supply, the best alternative for meeting the nutritional needs of the preterm infant is donor human milk (77, 78). Pasteurization of donor milk is mandatory in order to destroys high-risk viruses and non-spore-forming bacteria. The method extensively applied by human milk bank is the Holder process (HoP) and commonly involves heating donor milk at 62.5° C (145°F) for 30 minutes (79). The two studies which had investigated the effect of HoP on miRNAs reported somewhat conflicting results, with one reporting no change in selected miRNAs (45) and the other reporting substantial degradation of all miRNAs (49). The latter study suggested that an alternative high-pressure pasteurization method may be more sparing on the miRNA content, and future research should investigate the changes after different pasteurization techniques.

Uptake experiments have been limited to ncRNAs associated with breast milk EVs. Three research groups studied human milk EV uptake as a part of their investigations into miRNAs (18, 39, 40, 47) and circRNAs (54). Whist each groups adopted protocols for antibody labeling of fixed EVs and subsequent visualization by fluorescently labeled secondary antibodies, none of the studies used “free-dye” controls in their uptake experiments. Free-dye controls can be used to exclude the possibility that the dye aggregates and form micelles that may create a false positive response if taken up in the cell line (80). It is also important to note that cell line experiments like these always have limitations due to their distance to the true physiological setting. A possible development of these experiments could be the use of intestinal organoids or colonic tissue mounted in using chambers. The use of chambers or transwells would allow studies of the uptake of EVs over the intestinal epithelium and the supernatant from the serosal/basal side of the experimental system could be used for further functional studies downstream, for example immune cell stimulations. A further limitation in the studies by Liao et al. (18) and Kahn et al. (40) is the lack of a “background control” for the CD9 labeling of the EVs, since the CD9 tetraspanin is a common membrane protein in many cell types. Additionally, neither of the studies addressed here included any controls for non-specific binding of the secondary antibodies used for visualizing the EV uptake. A system for visualizing the uptake of EVs and their miRNA cargo on basis of GFP labeling of EV membrane proteins or miRNA coupled proteins such as RISC or Ago may be warranted.

In addition to specific functional properties investigated in the uptake experiments, *in silico* target and functional annotation analysis was used in most of the sequencing articles. The disparities in number of predicted gene targets (between 19 and 50 300) could be explained by several factors, e.g., the dataset used (i.e., a higher number of miRNAs included will result in more targets), the differences in the prediction tools used by the authors (i.e., the tools adopt similar approaches but not exactly the same criteria to predict miRNA and mRNA interactions), and the choices regarding thresholds and underlying statistics in these tools may contribute to the variation in the reported results. Specific details were rarely included, and some authors failed to state which version of the functional prediction database was used. With rapidly evolving functional prediction databases, reporting the database version provides important information for reproducibility and understanding of parameters used in target analyses. For example, new updates of TargetScan include new features, which improve the prediction accuracy (81–85). The same principle also applies for the other target prediction software, where each new version in general improves the prediction. Additionally, all target analysis data are solely based on computer prediction and none of the studies have included an experimental validation specifically investigating the role of the ncRNA on their predicted targets. The issues described above should be carefully considered when interpreting results and drawing conclusion from these studies. Nonetheless, the fact that the studies show similar target genes supports a certain degree of accuracy in their functional prediction. With the described functional predictions ranging from metabolic and biosynthetic processes to signaling pathways, cellular adhesion, communication, growth, and differentiation, it is difficult to infer the overall effects of breast milk ncRNAs, and further functional experiments are required.

### Factors Associated With miRNA Profile

The majority of included studies conducted some form of comparative analysis, yet it is difficult to draw confident conclusions from the published results due to the low number of participants in most studies, and because few studies have assessed the influence of the same factors on the same miRNAs. Lactational stage and milk fraction were the most widely examined factors. Broadly speaking, highly expressed miRNAs appear to be reasonably stable across lactational stages, yet differential expression was reported in miRNA with lower expression levels and two studies reported overall differences in the miRNA profile using multivariate analysis techniques. Similarly, many of the highly expressed miRNAs were found across all milk fractions. Whilst individual studies reported differences in the abundance of specific miRNAs between milk fractions, there was little consistency between studies. Furthermore, it is unclear whether miRNAs from a particular milk fraction are more or less biologically available to the infant who ultimately consumes all fractions. Experimental investigations into the biological availability of miRNAs in different milk fractions could guide milk processing choices in future studies.

The absolute quantity of miRNAs from each milk fraction is also likely to be an important consideration with respect to their biological functions as both physiological availability and quantity will affect the extent of their potential functions. Skim milk is the largest fraction by volume and may therefore represent the largest source of miRNAs to the infant, even though the concentration of miRNAs is reportedly higher in the cellular or lipid fractions (32, 35). Studies comparing milk fractions should therefore aim to measure the total quantity of miRNA between fractions. This could be captured by protocols starting with a fixed volume of whole milk and extracting all RNAs from each fraction, or by measuring the relative volume of each milk fraction so that the concentration found in whole milk can be estimated. This distinction between miRNA concentrations calculated per milk fraction or whole milk volume may explain the somewhat contradictory findings of Alsaweed and coauthors (32, 35) which suggest that the cellular fraction has the highest concentration of miRNA, and those from Qin et al. (57) who found the highest concentration of 11 miRNAs in the skim milk fraction. As noted earlier, the concentrations of miRNA from the cellular fraction in the studies by Alsaweed and coauthors are reported as nanograms per million cells and this is difficult to directly compare with the concentrations in the skim and lipid fractions which are measured in nanograms per milliliter. The methods described in Qin et al. (57) are insufficient to determine if their findings can be interpreted as an indication of the relative amount of these miRNAs received from each milk fraction. Since Qin et al. (57) used the snRNA Snord95 as an endogenous control to calculate the relative expression of the miRNAs, differences in Snord95 concentrations between milk fractions would also have affected their results. That said, the authors state that there was minimal variation in expression of Snord95 between participants which presumably implies they observed minimal variability between milk fractions as well as participants. Another challenge in the comparison of miRNAs and other ncRNAs in milk fractions is the choice of RNA extraction kit. As demonstrated by Alsaweed et al. (32), the yield and quality of RNAs extraction from each milk fraction varies between kits. Again, it would have been beneficial to compare these extraction kits with respect to the yield per mL of whole milk in order to remove the influence of starting and elution volumes on the final RNA quantity and concentration. Nonetheless, this work raises an interesting dilemma: what creates a greater bias in the miRNA profile, using an RNA extraction kit which is less optimal for the specific milk fraction or using different RNA extraction kits in the comparison of different milk fractions? There is certainly evidence that different RNA extraction kits result in distinct miRNA profiles (86, 87) and this should be kept in mind when interpreting the results comparing the lipid and cellular fraction in Alsaweed et al. (35). What remains unclear, is whether a greater bias is introduced by using a kit which is suboptimal for one or both milk fractions.

Turning our attention to other factors which may influence the miRNAs in human milk, there is currently insufficient evidence to conclusively identify maternal or infant factors associated with the abundance of specific miRNAs or the overall miRNA profile. The studies included in this reviewed considered maternal age, parity, BMI, stress, and smoking or smoke exposure, as well as gestational age and infant sex, growth, and eczema. It is biologically plausible that all of the factors could be associated with the human milk miRNA profile, and this should be investigated further in larger studies. Additionally, the incorporation of multivariate and dimension reduction techniques in the analysis of high-throughput data may lead to greater insights into the interplay between miRNAs, how they cluster together and exert common functions, and how they related to maternal and infant characteristics or infant health. Ultimately, these analyses should strive to incorporate other ncRNAs and bioactive components in human milk and seek opportunities for translational research with experimental and low-throughput validation studies. The recent article investigating gestation age and circRNAs (54) is a welcome addition to the field and should be followed-up with other studies to confirm their findings.

### Methodological Considerations Within Studies

Heterogeneity, lack of standardisation of sample collection and processing within and between studies is a common challenge in breast milk research (64–66). The studies included in this review were no exception and, like others before us, we identified a need for greater standardisation and clearer reporting of sample collection and processing methods (64, 66). There is currently insufficient evidence to determine if it is particularly important to standardise certain aspects of the collection and processing procedures when analysing ncRNAs, such as the time of day for collection, volume of milk, or the speed and duration of centrifugation. It is also unclear whether ncRNAs from different milk fraction are more or less biologically available, although the experimental stability and uptake experiments have focussed on milk EVs.

Several of the included studies claim to have isolated or enriched for milk “exosomes” – EVs originating from multivesicular bodies with a general size of 30-150nm (88). The recently updated ‘minimal information for studies of extracellular vesicles 2018’ (MISEV2018) guidelines warns against assigning EVs to a specific biogenesis pathway and points out the need for substantial characterization of the vesicles in order to claim that “exosomes” are specifically studied (89). Since most of the studies included in this review lack this confirmatory characterization, we have chosen to refer to this milk fraction merely as extracellular vesicles. Reliable and efficient isolation of EVs from complex biological matrices is important to ensure reproducibility and accurate analyses of EV content and function. There are five EV isolation techniques that has been developed based on: differential ultracentrifugation, density gradients, immunoaffinity capture, microfluidics, and precipitation. Each of the methods utilizes different vesicle traits in order to isolate them: density, shape, size, and surface proteins (88). In this review, a majority of the studies (18, 19, 39, 40, 43, 48, 55) used ExoQuick to isolate their vesicles, while three (14, 50, 54) used ultracentrifugation protocols, three (36, 41, 56) used the membrane-affinity based capture ExoEasy Maxi kit and one study used separation by antibody coated magnetic beads (14). The ultracentrifugation method is regarded as a fairly robust method for EV isolation whilst precipitation methods (such as ExoQuick), although easy to use, can easily result in contamination from non-EV structures (88). Regardless of the method of isolation, it is recommended that the obtained EV fraction be validated through analysis of EV-associated components and checking for non-vesicular components. Specifically, the MISEV2018 guidelines recommend that each preparation of EVs should be 1) defined by quantitative measures of the EV source (e.g., number of cells or volume of biofluid), 2) characterized by abundance of EVs, 3) tested for presence of EV-associated components (e.g., tetraspanins and flotillin), and 4) tested for presence of non-vesicular components (e.g., calnexin). To report how the samples have been stored would also be of interest, as this could affect stability, aggregation, and number of EVs (89). Considering the above-stated issues, the results from studies investigating EV-associated miRNAs without validation of the EV fraction should be interpreted with caution. Whilst these studies provide insights into the miRNAs ingested by breastfed infants, the reported miRNA profiles may not solely reflect EV-associated miRNAs. This is a particular concern for studies using precipitations methods, which are known, for example, to also capture extracellular and extravesicular protein-miRNA complexes (90).

After processing the milk samples, there exists a multitude of alternative approaches to RNA isolation, RNA quantification and analysis of the results. Few head-to-head assessments of methodological choices in the analysis of miRNAs in breast milk have been published. Starting with RNA isolation, Alsaweed et al. (32) analyzed the influence of eight different RNA isolation kits on the quantity and quality of RNA exaction, and proportion of miRNA length RNAs. No single RNA extract kit was found to be superior on all accounts, however the authors balanced the quantity and quality of the extracted RNA and judged miRNeasy mini, miRCURY Biofluids and miRCURY Cell & Plant kits as the most effective extraction kits for cell, lipids, and skim milk fractions, respectively. However, it is worth noting that this study did not standardize the input volume or the elution volume, nor did they include an exogenous control for technical variability. It is therefore unclear if higher RNA concentrations result from a larger volume of milk or from smaller elution volumes rather than the efficiency of the kit itself. The use of a known concentration of exogenous control would also have allowed the investigation of recovery of this particular miRNA by each kit. Nonetheless, we get an indication that extraction kits can affect the quantity and quality of total RNA and also the proportion of small RNAs. Whilst the influence of extraction kit on the specific miRNAs or the overall profile in human milk was not investigated, others have reported that the choice of both RNA extraction and library preparation kits influences miRNA analyses in plasma samples (86, 87). Library preparation kit, in particular, was found to have a greater effect on the resulting miRNA analyses in RNA-seq studies (86).

The quantification of RNA included qPCR and RNA-seq based methods. Originally developed for gene expression analysis, both methods are valuable assays in the investigation of small RNAs and start with principally similar chemical processes which convert the RNAs to cDNA. Although untested in human milk, previous studies have indicated that the choice of qPCR method (91, 92) appears to influence the abundance of specific miRNA, as can the library preparation kit in RNA-seq (86, 93). Furthermore, each library preparation kit employs different size selection routines and some include optional additional steps, such as the previously used Tobacco Acid Pyrophosphatase (TAP) incubation step in the ScriptMiner™ protocol (94, 95). This optional step modified the 5’ end of the RNAs to allow adapter ligation to 5’-capped and 5’-triphosphyorylated RNAs in addition to 5’ monophosphate RNAs. The majority of mammalian miRNAs have a single phosphate group at the 5’ end which means that this additional step would reduce the proportion of miRNA relative to other small RNAs. On the other hand, 5’ adaptors which do not ligate to other 5’ ends would result in a biased representation of the total small RNA profile (96) and would fail to reliably identify any miRNAs with capped 5’ ends (97). Similarly, the range of RNA lengths captured in the size selection steps will influence the proportion of miRNAs and the possibility to detect other ncRNAs. For example, the relatively wide size selection for human samples in van Herwijnen et al. (50), may be one of the reasons why they found that only 0.6% of the reads mapped to mature miRNAs.

Following the laboratory analysis, the methods used for normalisation and statistical analyses varied. The question of normalizing strategies in qPCR quantification of miRNA data is highly debated and the lack of endogenous “house-keeping” miRNAs pose a challenge for the field as a whole. The purpose of an internal control is to correct for biological and technical variabilities, meaning it is important for accurate interpretation of the results. Different normalization strategies were used in the studies, with the most common ones being the endogenous RNAs U-snRNAs (RNU6, RNU44 and RNU48) or synthesized exogenous miRNAs, and they all have different limitations that should be taken into consideration. The most frequently used endogenous normalization factor within the field, RNU6, is not a miRNA and therefore has different biochemical properties. For example, the molecule is longer, which might result in different efficiency of the extraction, the cDNA synthesis, and the PCR reaction, compared to the miRNAs in the sample. It has also been highlighted that the U-snRNAs are less chemically stable than miRNAs and the instability of their expression levels is also problematic when they are used as an endogenous control (98). On the other hand, the addition of known concentrations of exogenous controls allows normalization which accounts for variation between samples due to technical differences, but will not capture biological varibilities (98). Furthermore, other normalization strategies where no endogenous control is being quantified can be used. The global mean method used by Bozack (36) is based on the mean expression value of all detected miRNAs in all samples, followed by relative expression to that value. Floris et al. (38) calculated the geometric mean of endogenous miRNAs in the samples and used the values as normalization factors. Lastly, several computer programs (e.g. NormFinder (99), RefFinder (100) and geNorm (101)) have been designed to identify potential reference genes based on the expression pattern in the samples. Applying these methods to available RNA-seq datasets may identify candidate reference miRNAs in breast milk research, guiding experiments to confirm a stable and validated set of reference miRNAs.

In addition to qPCR studies, normalization is also an important source of variability between the RNA-seq studies. The most common normalization method for RNA-seq studies was the calculation of reads per million. However, it varied as to whether the reads were normalized to total clean reads (33, 35) or reads mapping to mature miRNA (19, 39, 42, 48, 49), and examination of the **Supplementary Files** cast doubt on how this procedure was performed in four articles (18, 33, 35, 40). A minority of articles used alternative or unclear normalization methods without a clear justification of why this approach was chosen (37, 43, 46, 50, 56). We note here that it is possible to include a spike-in synthetic RNA to help in the assessment of library quality and normalization, however this is not established as a gold standard method, remains relatively uncommon, and was not reported in any of the included articles. Regardless of the use of spike-in exogenous miRNAs, normalization to relative expression levels is required and this was done in all RNA-seq studies. Selected results from RNA-seq and microarray studies were often, but not always, validated using qPCR. These technical validations represent good practice and has been considered a gold standard for RNA-seq and microarray analyses. However, it is equally important that findings are validated in a new set of samples from a new study population (102). Beyond the laboratory analyses, there was also a range of statistical approaches employed in the analysis of both qPCR and RNA-seq studies. Particularly for RNA-seq studies, approaches which fail to account for multiple hypothesis testing involve a high risk of falsely identified differentially expressed miRNAs.

All the methodological challenges and heterogeneity between studies described here are important considerations and we hope to see greater standardization of methods as the field progresses. Nonetheless, for studies which have used consistent methods in the collection, processing, and analysis for all milk samples, we can assume that the comparisons within these studies are valid. On the other hand, the results from studies which appear to have employed different methods for different groups of samples should be interpreted cautiously (32, 34, 35, 37, 43).

### Strengths and Limitations of Systematic Review

The main strength of this review is the systematic approach used to identify all studies examining ncRNA species in breast milk of healthy lactating mothers. In planning the review, we were aware that some sequencing studies have reported a relatively low proportion of miRNAs, even when the sequencing protocol has focused on small RNAs. We therefore aimed to identify all studies investigating any of the ncRNA. Although we identified only two studies investigating ncRNAs other than miRNAs, we were also able to highlight the lack of knowledge around other ncRNA species. Another strength is that we have set out to provide a comprehensive summary covering both the endogenous miRNA profile, their stability and potential functions, in addition to evidence for maternal and infant characteristics affecting the abundance of ncRNAs and their associations with child health. Our systematic approach also included an assessment of study quality and potential biases, which provides an important revision of methodological challenges. When conducting this quality assessment, we took into consideration that this field is relatively new. We accepted that study aims could be broad questions in new fields and we placed a greater focus on clarity of reporting. Nonetheless, our systematic assessment of study quality highlights some methodological aspects which can be improved in many studies, and this is a strength of our review.

At the same time, our review faces some limitations. Heterogeneity between the studies meant that we could only provide a narrative summary of the current evidence. We also specified that only human studies were eligible for inclusion in this review. We appreciate that the high degree of conservation of miRNAs, and even milk-related miRNAs, among mammals means that there is a wealth of evidence from animal studies which was beyond the scope of this review. A recent review on the functional roles of milk EVs provides a comprehensive summary of current evidence from *in vitro*, human and animal experiments on the functional roles of milk EVs, looking at both EV-associated miRNAs and other bioactive components in milk EVs (103). Future reviews could also include evidence spanning *in vitro*, human and animal studies of miRNAs in other milk fractions and other ncRNAs. Factors associated with miRNA profile

The majority of included studies conducted some form of comparative analysis, yet it is difficult to draw confident conclusions from the published results due to the low number of participants in most studies, and because few studies have assessed the influence of the same factors on the same miRNAs. Lactational stage and milk fraction were the most widely examined factors. Broadly speaking, highly expressed miRNAs appear to be reasonably stable across lactational stages, yet differential expression was reported in miRNA with lower expression levels and two studies reported overall differences in the miRNA profile using multivariate analysis techniques. Similarly, many of the highly expressed miRNAs were found across all milk fractions. Whilst individual studies reported differences in the abundance of specific miRNAs between milk fractions, there was little consistency between studies. Furthermore, it is unclear whether miRNAs from a particular milk fraction are more or less biologically available to the infant who ultimately consumes all fractions. Experimental investigations into the biological availability of miRNAs in different milk fractions could guide milk processing choices in future studies.

The absolute quantity of miRNAs from each milk fraction is also likely to be an important consideration with respect to their biological functions as both physiological availability and quantity will affect the extent of their potential functions. Skim milk is the largest fraction by volume and may therefore represent the largest source of miRNAs to the infant, even though the concentration of miRNAs is reportedly higher in the cellular or lipid fractions (32, 35). Studies comparing milk fractions should therefore aim to measure the total quantity of miRNA between fractions. This could be captured by protocols starting with a fixed volume of whole milk and extracting all RNAs from each fraction, or by measuring the relative volume of each milk fraction so that the concentration found in whole milk can be estimated. This distinction between miRNA concentrations calculated per milk fraction or whole milk volume may explain the somewhat contradictory findings of Alsaweed and coauthors (32, 35) which suggest that the cellular fraction has the highest concentration of miRNA, and those from Qin et al. (57) who found the highest concentration of 11 miRNAs in the skim milk fraction. As noted earlier, the concentrations of miRNA from the cellular fraction in the studies by Alsaweed and coauthors are reported as nanograms per million cells and this is difficult to directly compare with the concentrations in the skim and lipid fractions which are measured in nanograms per milliliter. The methods described in Qin et al. (57) are insufficient to determine if their findings can be interpreted as an indication of the relative amount of these miRNAs received from each milk fraction. Since Qin et al. (57) used the snRNA Snord95 as an endogenous control to calculate the relative expression of the miRNAs, differences in Snord95 concentrations between milk fractions would also have affected their results. That said, the authors state that there was minimal variation in expression of Snord95 between participants which presumably implies they observed minimal variability between milk fractions as well as participants. Another challenge in the comparison of miRNAs and other ncRNAs in milk fractions is the choice of RNA extraction kit. As demonstrated by Alsaweed et al. (32), the yield and quality of RNAs extraction from each milk fraction varies between kits. Again, it would have been beneficial to compare these extraction kits with respect to the yield per mL of whole milk in order to remove the influence of starting and elution volumes on the final RNA quantity and concentration. Nonetheless, this work raises an interesting dilemma: what creates a greater bias in the miRNA profile, using an RNA extraction kit which is less optimal for the specific milk fraction or using different RNA extraction kits in the comparison of different milk fractions? There is certainly evidence that different RNA extraction kits result in distinct miRNA profiles (86, 87) and this should be kept in mind when interpreting the results comparing the lipid and cellular fraction in Alsaweed et al. (35). What remains unclear, is whether a greater bias is introduced by using a kit which is suboptimal for one or both milk fractions.

Turning our attention to other factors which may influence the miRNAs in human milk, there is currently insufficient evidence to conclusively identify maternal or infant factors associated with the abundance of specific miRNAs or the overall miRNA profile. The studies included in this reviewed considered maternal age, parity, BMI, stress, and smoking or smoke exposure, as well as gestational age and infant sex, growth, and eczema. It is biologically plausible that all of the factors could be associated with the human milk miRNA profile, and this should be investigated further in larger studies. Additionally, the incorporation of multivariate and dimension reduction techniques in the analysis of high-throughput data may lead to greater insights into the interplay between miRNAs, how they cluster together and exert common functions, and how they related to maternal and infant characteristics or infant health. Ultimately, these analyses should strive to incorporate other ncRNAs and bioactive components in human milk and seek opportunities for translational research with experimental and low-throughput validation studies. The recent article investigating gestation age and circRNAs (54) is a welcome addition to the field and should be followed-up with other studies to confirm their findings.

### Future Research and Challenges

Through this systematic review, we have identified a number of challenges and areas for future research. There remain many unanswered questions around the basic biology and mechanisms of ncRNA in human milk.

First, it is clear that only a fraction of the ncRNAs present in human breast milk have been characterized and future studies should take a focused look at some of these other ncRNA species which are also implicated in regulation of gene expression. Along the same lines, further research is needed to understand how different ncRNAs are released into milk, and whether different physiological processes control their release into different milk fractions. Second, future studies should seek to understand how ncRNAs are taken up from milk and to what extend they pass the intestinal barrier and reach potential target organs in humans. The use of organoids is likely to provide a more physiologically relevant *in vitro* model for future uptake and functional experiments and can supplement experimental *in vivo* animal models. Third, the physiological function of different ncRNAs should also be further investigated, including studies into different milk fractions. To date, the experiments into the stability and uptake have focused on miRNAs in EVs and it is less clear if other miRNAs and ncRNAs present in the lipid or cellular fractions are equally stable or biologically active. Fourth, high-throughput studies should endeavor to incorporate analysis and functional prediction methods which aim to identify the collective effect of human milk RNAs, such as multivariate and dimensions reductions analysis techniques. Fifth, there a need for larger studies into the effect of specific ncRNAs in infants and to determine if maternal health, environmental or nutritional factors can influence their abundance.

Whilst these are lines of exciting future research, and by no means a complete list of the unanswered questions, it is also important to address some of the challenges in the field. Heterogeneity and lack of standardisation of methods makes it difficult to confidently combine results from different studies. This heterogeneity stems from differences in all methodological stages from sample collection, storage and processing of samples, RNA isolation, library preparation for RNA-seq studies, and to the multiple choices associated with both PCR and RNA-seq as methods of quantification. Differences in the normalisation strategy, the bioinformatic pipeline and the statistical analyses also introduce heterogeneity that makes comparisons between studies difficult. The International Society for Extracellular Vesicles (ISEV) have established a Rigor & Standardization Subcommittee with a Task Force working on the standardisation of methods for research into milk EVs (104). Along the same lines, a collaborative, evidence-based guideline for “best practice” for research into ncRNAs from all milk fractions is warranted and funding bodies should support efforts to investigate the consequences of different methodological choices in human milk research in both ncRNAs and other bioactive components. Studies aimed to identify appropriate endogenous controls for normalisation in qPCR experiments and head-to-head comparisons of sequencing technologies are particularly relevant in the field of ncRNA. Whilst it may be unnecessary or unrealistic to standardise all these sources of variation, there is a need for greater transparency in the reporting of each of these methodological stages. For example, we noted that many studies described RNA isolation or sequencing as performed “according to manufacturer instructions”, yet the protocols from manufacturers often include options in steps and the choices made around these could influence the final results and how easily they can be compared to other studies. These sorts of methodological details could be provided in online **Supplementary Files** which have become more widely used in recent years. Finally, many of the included studies are small and underpowered to confidently identify differences in ncRNA expression, something which has a negative impact on the reproducibility of any findings. Sample size calculations are difficult in emerging fields since the parameters required for these calculations are largely unknown and can be particularly complicated in high-throughput experiments. As the field progresses, we hope to see new studies drawing on current evidence and a greater use of pilot studies to identify appropriate sample sizes, particularly when the experimental protocols differ from previous studies.

## Conclusions

Human breast milk contains high quantities of RNA which, at a minimum, includes miRNAs, lncRNAs, circRNAs, piRNAs, snoRNAs, snRNAs, and fragmented tRNAs and rRNAs. Among these ncRNAs, it is only miRNAs which have been specifically investigated in more than a single study. The presence of miRNAs has been confirmed in the skim milk, cellular and lipid fraction of human breast milk, as well as encapsulated in milk EVs. Most studies exploring the overall miRNA profile found that the top 10 miRNAs accounted for ~60 to 80% of all miRNA reads, and often included miR-148a-3p, miR-30a/d-5p, miR-22-3p, miR-146b-5p, miR-200a/c-3p, and the 5p end of the let-7 miRNA. These miRNAs were observed to be highly expressed in all milk fractions, and in both fresh and frozen samples. At the same time, some studies report quite divergent miRNAs profiles which highlights the need for careful investigation of the influence of methodological choices and to identify what biases are introduced or mitigated by these choices.

Experimental evidence suggests that EV-associated miRNAs in human milk are stable under a range of harsh conditions, including those mimicking the infant gut, and that they are taken up by intestinal epithelial cells under *in vitro* experiments. Based on mRNA target predictions, human milk miRNAs have a wide potential range of functions. *In vitro* experiments have confirmed specific gene expression regulatory effects for individual miRNAs and circRNAs, but the collective effect of ncRNAs in human milk still needs to be investigated. Similarly, there is a need for further investigations of the influence of lactational, maternal and infant characteristics on human milk miRNAs, as well as examining their role in infant health and development.

## Data Availability Statement

The original contributions presented in the study are included in the article/**Supplementary Material**. Further inquiries can be directed to the corresponding author.

## Author Contributions

LT, EA, MS, and EC contributed to the conception and design of the study. SP also contributed to the design and conducted the literature search. LT, EA, LJ, and MS extracted the data, and LT and MS completed and assessed the quality. EC, KC, and PS contributed expert advice in the interpretation of the results. All authors contributed to the article and approved the submitted version.

## Conflict of Interest

The authors declare that the research was conducted in the absence of any commercial or financial relationships that could be construed as a potential conflict of interest.

## Publisher’s Note

All claims expressed in this article are solely those of the authors and do not necessarily represent those of their affiliated organizations, or those of the publisher, the editors and the reviewers. Any product that may be evaluated in this article, or claim that may be made by its manufacturer, is not guaranteed or endorsed by the publisher.
