# Supplementary material for: Non-Coding RNAs in Human Breast Milk: A Systematic Review
Source: Front Immunol. 2021 Sep 1;12:725323. doi: 10.3389/fimmu.2021.725323 (PMC8440964; doi:10.3389/fimmu.2021.725323)
Supplement: Supplementary file 1 [file DataSheet_1.pdf]

Supplementary Material

Non-coding RNAs in Human Breast Milk: A Systematic Review

Lina Tingö[1,2]†, Emelie Ahlberg[1]†, Lovisa Johansson[3], Sindre Andre Pedersen[4], Konika Chawla[5,6.7], Pål Sætrom[5,6,7,8], Erika Cione[9], Melanie Rae Simpson[10,11*]

†These authors have contributed equally to this work and share first authorship

[1] Department of Biomedical and Clinical Sciences, Linköping University, Linköping, Sweden
[2] School of Medical Sciences, Örebro University, Örebro, Sweden
[3] Division of Neurobiology, Department of Biomedical and Clinical Sciences, Linköping University, Linköping, Sweden
[4] Library Section for Medicine and Health Sciences, NTNU Norwegian University of Science and Technology, Trondheim, Norway
[5] Department of Clinical and Molecular Medicine, NTNU Norwegian University of Science and Technology, Trondheim, Norway
[6] Bioinformatics core facility - BioCore, NTNU Norwegian University of Science and Technology, Trondheim, Norway
[7] K.G. Jebsen Center for Genetic Epidemiology, NTNU Norwegian University of Science and Technology, Trondheim, Norway
[8] Department of Computer Science, NTNU Norwegian University of Science and Technology, Trondheim, Norway
[9] Department of Pharmacy, Health and Nutritional Sciences, University of Calabria, Rende, Italy
[10] Department of Public Health and Nursing, NTNU Norwegian University of Science and Technology, Trondheim, Norway
[11] Clinic of Laboratory Medicine, St Olavs Hospital, Trondheim, Norway

Corresponding author: Melanie Rae Simpson, melanie.simpson@ntnu.no

---

Contents of supplementary material

1 Literature search details in the various databases 3
1.1 Ovid MEDLINE(R) ALL <1946 to November 13, 2020> 3
1.2 Cochrane Library 6
1.3 Embase <1974 to 2020 November 13> 8
1.4 Web of Science 11
2 Approach to assessment of quality 13
3 Supplementary results 14
3.1 Table S1: Description of read proportions in RNAseq studies 14
3.2 Table S2: Top 10 novel miRNA candidates described in human milk 15
3.3 Table S3: Quality assessment of included studies 16
3.4 Table S4: Statistical approach in studies employing targeted PCR only 17
3.5 Table S5: Statistical approach in studies employing RNAseq or TaqMan OpenArray 18

1 Literature search details in the various databases

1.1 Ovid MEDLINE(R) ALL <1946 to November 13, 2020>

1	[Breast milk concept]
2	exp Colostrum/
3	exp Breast Feeding/
4	exp Lactation/
5	Milk, human/
6	(breastmilk or breast-milk or mothersmilk or mothers-milk or ((breast or human? or maternal or mother? or woman) adj2 milk)).ti,ab,kw.
7	(breastfe* or breast-fe* or (breast adj2 (feed* or fed))).ti,ab,kw.
8	(colostrum* or colostral* or colostrium or foremilk or fore-milk or hindmilk or hind-milk).ti,ab,kw.
9	(lactation or breast-secretion* or milk-release or ((breast or milk) adj2 (secretion* or release))).ti,ab,kw.
10	or/2-9
11	[Non-coding RNA concept]
12	RNA, Antisense/
13	exp MicroRNAs/
14	exp RNA, Small Interfering/
15	exp RNA, Nuclear/
16	exp RNA, Small Nuclear/
17	exp RNA, Small Nucleolar/
18	exp RNA, Ribosomal/
19	exp RNA, Transfer/
20	exp RNA, Untranslated/
21	RNA, Catalytic/
22	RNA, Long Noncoding/
23	exp RNA, Small Untranslated/
24	MicroRNAs/
25	Circulating MicroRNA/
26	RNA, Guide/
27	RNA, Small Cytoplasmic/
28	RNA, Small Interfering/
29	RNA, Spliced Leader/
30	exp Untranslated Regions/
31	noncoding-ribonucleic-acid?.ti,ab,kw.
32	noncoding-RNA?.ti,ab,kw.
33	non-conding-ribonucleic-acid?.ti,ab,kw.
34	non-conding-RNA?.ti,ab,kw.
35	nonprotein-coding-ribonucleic-acid?.ti,ab,kw.
36	nonprotein-coding-RNA?.ti,ab,kw.
37	non-protein-coding-ribonucleic-acid?.ti,ab,kw.
38	non-protein-coding-RNA?.ti,ab,kw.
39	uncoding-ribonucleic-acid?.ti,ab,kw.
40	uncoding-RNA?.ti,ab,kw.
41	non-Messenger-ribonucleic-acid?.ti,ab,kw.
42	non-messenger-RNA?.ti,ab,kw.
43	junk-ribonucleic-acid?.ti,ab,kw.
44	junk-RNA?.ti,ab,kw.
45	garbage-ribonucleic-acid?.ti,ab,kw.
46	garbage-RNA?.ti,ab,kw.
47	untranslated-ribonucleic-acid?.ti,ab,kw.

48 untranslated-RNA?.ti,ab,kw.
49 nontranslated-RNA?.ti,ab,kw.
50 non-translated-ribonucleic-acid?.ti,ab,kw.
51 non-translated-RNA?.ti,ab,kw.
52 long-noncoding-ribonucleic-acid?.ti,ab,kw.
53 long-noncoding-RNA?.ti,ab,kw.
54 long-non-coding-RNA?.ti,ab,kw.
55 long-non-coding-ribonucleic-acid?.ti,ab,kw.
56 long-non-protein-coding-ribonucleic-acid?.ti,ab,kw.
57 long-non-protein-coding-RNA?.ti,ab,kw.
58 long-untranslated-ribonucleic-acid?.ti,ab,kw.
59 long-untranslated-RNA?.ti,ab,kw.
60 long-non-translated-ribonucleic-acid?.ti,ab,kw.
61 long-non-translated-RNA?.ti,ab,kw.
62 long-intergenic-non-protein-coding-ribonucleic-acid?.ti,ab,kw.
63 long-intergenic-non-protein-coding-RNA?.ti,ab,kw.
64 lincRNA?.ti,ab,kw.
65 linc-RNA?.ti,ab,kw.
66 lncRNA?.ti,ab,kw.
67 lnc-RNA?.ti,ab,kw.
68 ncRNA?.ti,ab,kw.
69 nc-RNA?.ti,ab,kw.
70 small-ribonucleic-acid?.ti,ab,kw.
71 small-RNA?.ti,ab,kw.
72 sRNA?.ti,ab,kw.
73 micro-ribonucleic-acid?.ti,ab,kw.
74 microRNA?.ti,ab,kw.
75 micro-RNA?.ti,ab,kw.
76 miRNA?.ti,ab,kw.
77 mi-RNA?.ti,ab,kw.
78 mirs.ti,ab,kw.
79 circulating-micro-ribonucleic-acid?.ti,ab,kw.
80 circulating-micro-RNA?.ti,ab,kw.
81 small-intefering-ribonucleic-acid?.ti,ab,kw.
82 small-intefering-RNA?.ti,ab,kw.
83 short-interfering-ribonucleic-acid?.ti,ab,kw.
84 short-interfering-RNA?.ti,ab,kw.
85 silencing-ribonucleic-acid?.ti,ab,kw.
86 silencing-RNA?.ti,ab,kw.
87 siRNA?.ti,ab,kw.
88 si-RNA?.ti,ab,kw.
89 trans-actins-siRNA?.ti,ab,kw.
90 tasiRNA?.ti,ab,kw.
91 tasi-RNA?.ti,ab,kw.
92 repeat-associated-ribonucleic-acid?.ti,ab,kw.
93 repeat-associated-RNA?.ti,ab,kw.
94 rasi-RNA?.ti,ab,kw.
95 rasiRNA?.ti,ab,kw.
96 piwi-interacting-ribonucleic-acid?.ti,ab,kw.

97 piwi-interacting-RNA?.ti,ab,kw.
98 piRNA?.ti,ab,kw.
99 pi-RNA?.ti,ab,kw.
100 short-haripin-ribonucleic-acid?.ti,ab,kw.
101 short-haripin-RNA?.ti,ab,kw.
102 small-hairpin-ribonucleic-acid?.ti,ab,kw.
103 small-hairpin-RNA?.ti,ab,kw.
104 sh-RNA?.ti,ab,kw.
105 shRNA?.ti,ab,kw.
106 small-scan-ribonucleic-acid?.ti,ab,kw.
107 small-scan-RNA?.ti,ab,kw.
108 scn-RNA?.ti,ab,kw.
109 scnRNA?.ti,ab,kw.
110 small-nucleolar-ribonucleic-acid?.ti,ab,kw.
111 small-nucleolar-RNA?.ti,ab,kw.
112 snoRNA?.ti,ab,kw.
113 sno-RNA?.ti,ab,kw.
114 small-nuclear-ribonucleic-acid?.ti,ab,kw.
115 small-nuclear-RNA?.ti,ab,kw.
116 snRNA?.ti,ab,kw.
117 sn-RNA?.ti,ab,kw.
118 small-cajal-body-specific-ribonucleic-acid?.ti,ab,kw.
119 small-cajal-body-specific-RNA?.ti,ab,kw.
120 scaRNA?.ti,ab,kw.
121 sca-RNA?.ti,ab,kw.
122 extracellular-ribonucleic-acid?.ti,ab,kw.
123 extracellular-RNA?.ti,ab,kw.
124 exosomal-ribonucleic-acid?.ti,ab,kw.
125 exosomal-RNA?.ti,ab,kw.
126 ex-RNA?.ti,ab,kw.
127 transfer-ribonucleic-acid?.ti,ab,kw.
128 transfer-RNA?.ti,ab,kw.
129 soluble-ribonucleic-acid?.ti,ab,kw.
130 soluble-RNA?.ti,ab,kw.
131 tRNA?.ti,ab,kw.
132 t-RNA?.ti,ab,kw.
133 tRF?.ti,ab,kw.
134 tRNA-derived-small-ribonucleic-acid?.ti,ab,kw.
135 tRNA-derived-small-RNA?.ti,ab,kw.
136 t-RNA-derived-small-RNA?.ti,ab,kw.
137 tsRNA?.ti,ab,kw.
138 ts-RNA?.ti,ab,kw.
139 ribosomal-ribonucleic-acid?.ti,ab,kw.
140 ribosomal-RNA?.ti,ab,kw.
141 ribosome-ribonucleic-acid?.ti,ab,kw.
142 ribosome-RNA?.ti,ab,kw.
143 rRNA?.ti,ab,kw.
144 r-RNA?.ti,ab,kw.
145 circular-ribonucleic-acid?.ti,ab,kw.
146 circular-RNA?.ti,ab,kw.
147 circRNA?.ti,ab,kw.

148 circ-RNA?.ti,ab,kw.
149 or/12-148
150 10 and 149

1.2 Cochrane Library

#1 MeSH descriptor: [Colostrum] explode all trees
#2 MeSH descriptor: [Breast Feeding] explode all trees
#3 MeSH descriptor: [Lactates] explode all trees
#4 MeSH descriptor: [Milk, Human] explode all trees
#5 (((breastmilk or breast-milk or mothersmilk or mothers-milk or ((breast or human? or maternal or mother? or woman) NEAR/2 milk)))):ti,ab,kw (Word variations have been searched)
#6 (breastfe* or breast-fe* or (breast NEAR/2 (feed* or fed))):ti,ab,kw (Word variations have been searched)
#7 (colostrum* or colostral* or colostrium or foremilk or fore-milk or hindmilk or hind-milk):ti,ab,kw (Word variations have been searched)
#8 (lactation or breast-secretion* or milk-release or ((breast or milk) NEAR/2 (secretion* or release))):ti,ab,kw (Word variations have been searched)
#9 #1 OR #2 OR #3 OR #4 OR #5 OR #6 OR #7 OR #8 25111
#10 MeSH descriptor: [RNA, Antisense] explode all trees
#11 MeSH descriptor: [MicroRNAs] explode all trees
#12 MeSH descriptor: [RNA, Small Interfering] explode all trees
#13 MeSH descriptor: [RNA, Nuclear] explode all trees
#14 MeSH descriptor: [RNA, Small Nuclear] explode all trees
#15 MeSH descriptor: [RNA, Small Nucleolar] explode all trees
#16 MeSH descriptor: [RNA, Ribosomal] explode all trees
#17 MeSH descriptor: [RNA, Transfer] explode all trees
#18 MeSH descriptor: [RNA, Untranslated] explode all trees
#19 MeSH descriptor: [RNA, Catalytic] explode all trees
#20 MeSH descriptor: [RNA, Long Noncoding] explode all trees
#21 MeSH descriptor: [RNA, Small Untranslated] explode all trees
#22 MeSH descriptor: [MicroRNAs] explode all trees
#23 MeSH descriptor: [Circulating MicroRNA] explode all trees
#24 MeSH descriptor: [RNA, Guide] explode all trees
#25 MeSH descriptor: [RNA, Small Cytoplasmic] explode all trees
#26 MeSH descriptor: [RNA, Small Interfering] explode all trees
#27 MeSH descriptor: [RNA, Spliced Leader] explode all trees
#28 MeSH descriptor: [Untranslated Regions] explode all trees
#29 (noncoding-ribonucleic-acid?):ti,ab,kw (Word variations have been searched)
#30 (noncoding-RNA?):ti,ab,kw (Word variations have been searched)
#31 (non-conding-ribonucleic-acid?):ti,ab,kw (Word variations have been searched)
#32 (non-conding-RNA?):ti,ab,kw (Word variations have been searched)
#33 (nonprotein-coding-ribonucleic-acid?):ti,ab,kw (Word variations have been searched)
#34 (nonprotein-coding-RNA?):ti,ab,kw (Word variations have been searched)
#35 (non-protein-coding-ribonucleic-acid?):ti,ab,kw (Word variations have been searched)
#36 (non-protein-coding-RNA?):ti,ab,kw (Word variations have been searched)
#37 (uncoding-ribonucleic-acid?):ti,ab,kw (Word variations have been searched)
#38 (uncoding-RNA?):ti,ab,kw (Word variations have been searched)
#39 (non-Messenger-ribonucleic-acid?):ti,ab,kw (Word variations have been searched)
#40 (non-messenger-RNA?):ti,ab,kw (Word variations have been searched)
#41 (junk-ribonucleic-acid?):ti,ab,kw (Word variations have been searched)
#42 (junk-RNA?):ti,ab,kw (Word variations have been searched)
#43 (garbage-ribonucleic-acid?):ti,ab,kw (Word variations have been searched)
#44 (garbage-RNA?):ti,ab,kw (Word variations have been searched)
#45 (untranslated-ribonucleic-acid?):ti,ab,kw (Word variations have been searched)
#46 (untranslated-RNA?):ti,ab,kw (Word variations have been searched)
#47 (nontranslated-RNA?):ti,ab,kw (Word variations have been searched)
#48 (non-translated-ribonucleic-acid?):ti,ab,kw (Word variations have been searched)
#49 (non-translated-RNA?):ti,ab,kw (Word variations have been searched)
#50 (long-noncoding-ribonucleic-acid?):ti,ab,kw (Word variations have been searched)
#51 (long-noncoding-RNA?):ti,ab,kw (Word variations have been searched)
#52 (long-non-coding-RNA?):ti,ab,kw (Word variations have been searched)

#53 (long-non-coding-ribonucleic-acid?):ti,ab,kw (Word variations have been searched)
#54 (long-non-protein-coding-ribonucleic-acid?):ti,ab,kw (Word variations have been searched)
#55 (long-non-protein-coding-RNA?):ti,ab,kw (Word variations have been searched)
#56 (long-untranslated-ribonucleic-acid?):ti,ab,kw (Word variations have been searched)
#57 (long-untranslated-RNA?):ti,ab,kw (Word variations have been searched)
#58 (long-non-translated-ribonucleic-acid?):ti,ab,kw (Word variations have been searched)
#59 (long-non-translated-RNA?):ti,ab,kw (Word variations have been searched)
#60 (long-intergenic-non-protein-coding-ribonucleic-acid?):ti,ab,kw (Word variations have been searched)
#61 (long-intergenic-non-protein-coding-RNA?):ti,ab,kw (Word variations have been searched)
#62 (lincRNA?):ti,ab,kw (Word variations have been searched)
#63 (linc-RNA?):ti,ab,kw (Word variations have been searched)
#64 (lncRNA?):ti,ab,kw (Word variations have been searched)
#65 (lnc-RNA):ti,ab,kw (Word variations have been searched)
#66 (ncRNA?):ti,ab,kw (Word variations have been searched)
#67 (nc-RNA?):ti,ab,kw (Word variations have been searched)
#68 (small-ribonucleic-acid?):ti,ab,kw (Word variations have been searched)
#69 (small-RNA?):ti,ab,kw (Word variations have been searched)
#70 (sRNA?):ti,ab,kw (Word variations have been searched)
#71 (micro-ribonucleic-acid?):ti,ab,kw (Word variations have been searched)
#72 (microRNA?):ti,ab,kw (Word variations have been searched)
#73 (micro-RNA?):ti,ab,kw (Word variations have been searched)
#74 (miRNA?):ti,ab,kw (Word variations have been searched)
#75 (mi-RNA?):ti,ab,kw (Word variations have been searched)
#76 (mirs):ti,ab,kw (Word variations have been searched)
#77 (circulating-micro-ribonucleic-acid?):ti,ab,kw (Word variations have been searched)
#78 (circulating-micro-RNA?):ti,ab,kw (Word variations have been searched)
#79 (small-intefering-ribonucleic-acid?):ti,ab,kw (Word variations have been searched)
#80 (small-intefering-RNA?):ti,ab,kw (Word variations have been searched)
#81 (short-interfering-ribonucleic-acid?):ti,ab,kw (Word variations have been searched)
#82 (short-interfering-RNA?):ti,ab,kw (Word variations have been searched)
#83 (silencing-ribonucleic-acid?):ti,ab,kw (Word variations have been searched)
#84 (silencing-RNA?):ti,ab,kw (Word variations have been searched)
#85 (siRNA?):ti,ab,kw (Word variations have been searched)
#86 (si-RNA?):ti,ab,kw (Word variations have been searched)
#87 (trans-actins-siRNA?):ti,ab,kw (Word variations have been searched)
#88 (tasiRNA?):ti,ab,kw (Word variations have been searched)
#89 (tasi-RNA?):ti,ab,kw (Word variations have been searched)
#90 (repeat-associated-ribonucleic-acid?):ti,ab,kw (Word variations have been searched)
#91 (repeat-associated-RNA?):ti,ab,kw (Word variations have been searched)
#92 (rasi-RNA?):ti,ab,kw (Word variations have been searched)
#93 (rasiRNA?):ti,ab,kw (Word variations have been searched)
#94 (piwi-interacting-ribonucleic-acid?):ti,ab,kw (Word variations have been searched)
#95 (piwi-interacting-RNA?):ti,ab,kw (Word variations have been searched)
#96 (piRNA?):ti,ab,kw (Word variations have been searched)
#97 (pi-RNA?):ti,ab,kw (Word variations have been searched)
#98 (short-haripin-ribonucleic-acid?):ti,ab,kw (Word variations have been searched)
#99 (short-haripin-RNA?):ti,ab,kw (Word variations have been searched)
#100 (small-hairpin-ribonucleic-acid?):ti,ab,kw (Word variations have been searched)
#101 (small-hairpin-RNA?):ti,ab,kw (Word variations have been searched)
#102 (sh-RNA?):ti,ab,kw (Word variations have been searched)
#103 (shRNA?):ti,ab,kw (Word variations have been searched)
#104 (small-scan-ribonucleic-acid?):ti,ab,kw (Word variations have been searched)
#105 (small-scan-RNA?):ti,ab,kw (Word variations have been searched)
#106 (scn-RNA?):ti,ab,kw (Word variations have been searched)
#107 (scnRNA?):ti,ab,kw (Word variations have been searched)
#108 (small-nucleolar-ribonucleic-acid?):ti,ab,kw (Word variations have been searched)
#109 (small-nucleolar-RNA?):ti,ab,kw (Word variations have been searched)
#110 (snoRNA?):ti,ab,kw (Word variations have been searched)
#111 (sno-RNA?):ti,ab,kw (Word variations have been searched)
#112 (small-nuclear-ribonucleic-acid?):ti,ab,kw (Word variations have been searched)
#113 (small-nuclear-RNA?):ti,ab,kw (Word variations have been searched)
#114 (snRNA?):ti,ab,kw (Word variations have been searched)
#115 (sn-RNA?):ti,ab,kw (Word variations have been searched)
#116 (small-cajal-body-specific-ribonucleic-acid?):ti,ab,kw (Word variations have been searched)
#117 (small-cajal-body-specific-RNA?):ti,ab,kw (Word variations have been searched)
#118 (scaRNA?):ti,ab,kw (Word variations have been searched)

#119 (sca-RNA?):ti,ab,kw (Word variations have been searched)
#120 (extracellular-ribonucleic-acid?):ti,ab,kw (Word variations have been searched)
#121 (extracellular-RNA?):ti,ab,kw (Word variations have been searched)
#122 (exosomal-ribonucleic-acid?):ti,ab,kw (Word variations have been searched)
#123 (exosomal-RNA?):ti,ab,kw (Word variations have been searched)
#124 (ex-RNA?):ti,ab,kw (Word variations have been searched)
#125 (transfer-ribonucleic-acid?):ti,ab,kw (Word variations have been searched)
#126 (transfer-RNA?):ti,ab,kw (Word variations have been searched)
#127 (soluble-ribonucleic-acid?):ti,ab,kw (Word variations have been searched)
#128 (soluble-RNA?):ti,ab,kw (Word variations have been searched)
#129 (tRNA?):ti,ab,kw (Word variations have been searched)
#130 (t-RNA?):ti,ab,kw (Word variations have been searched)
#131 (tRF?):ti,ab,kw (Word variations have been searched)
#132 (tRNA-derived-small-ribonucleic-acid?):ti,ab,kw (Word variations have been searched)
#133 (tRNA-derived-small-RNA?):ti,ab,kw (Word variations have been searched)
#134 (t-RNA-derived-small-RNA?):ti,ab,kw (Word variations have been searched)
#135 (tsRNA?):ti,ab,kw (Word variations have been searched)
#136 (ts-RNA?):ti,ab,kw (Word variations have been searched)
#137 (ribosomal-ribonucleic-acid?):ti,ab,kw (Word variations have been searched)
#138 (ribosomal-RNA?):ti,ab,kw (Word variations have been searched)
#139 (ribosome-ribonucleic-acid?):ti,ab,kw (Word variations have been searched)
#140 (ribosome-RNA?):ti,ab,kw (Word variations have been searched)
#141 (rRNA?):ti,ab,kw (Word variations have been searched)
#142 (r-RNA?):ti,ab,kw (Word variations have been searched)
#143 (circular-ribonucleic-acid?):ti,ab,kw (Word variations have been searched)
#144 (circular-RNA?):ti,ab,kw (Word variations have been searched)
#145 (circRNA?):ti,ab,kw (Word variations have been searched)
#146 (circ-RNA?):ti,ab,kw (Word variations have been searched)
#147 #10 OR #11 OR #12 OR #13 OR #14 OR #15 OR #16 OR #17 OR #18 OR #19 OR #20 OR #21 OR #22 OR #23 OR #24 OR #25 OR #26 OR #27 OR #29 OR #30 OR #31 OR #32 OR #33 OR #34 OR #35 OR #36 OR #37 OR #38 OR #39 OR #40 OR #41 OR #41 OR #42 OR #43 OR #44 OR #45 OR #46 OR #47 OR #48 OR #49 OR #50 OR #51 OR #52 OR #53 OR #54 OR #55 OR #56 OR #57 OR #58 OR #59 OR #60 OR #61 OR #62 OR #63 OR #64 OR #65 OR #66 OR #67 OR #68 OR #69 OR #70 OR #71 OR #72 OR #73 OR #74 OR #75 OR #76 OR #77 OR #78 OR #79 OR #80 OR #81 OR #82 OR #83 OR #84 OR #85 OR #86 OR #87 OR #88 OR #89 OR #90 OR #91 OR #92 OR #93 OR #94 OR #95 OR #96 OR #97 OR #98 OR #99 OR #100 OR #101 OR #102 OR #103 OR #104 OR #105 OR #106 OR #107 OR #108 OR #109 OR #110 OR #111 OR #112 OR #113 OR #114 OR #115 OR #116 OR #117 OR #118 OR #119 OR #120 OR #121 OR #122 OR #123 OR #124 OR #125 OR #126 OR #127 OR #128 OR #129 OR #130 OR #131 OR #131 OR #132 OR #133 OR #134 OR #135 OR #136 OR #137 OR #138 OR #139 OR #140 OR #141 OR #141 OR #142 OR #142 OR #143 OR #144 OR #145 OR #146
#148 #9 AND #147

1.3 Embase <1974 to 2020 November 13>

1 [Breast milk concept]
2 exp Colostrum/
3 exp Breast Feeding/
4 exp Lactation/
5 Breast milk/
6 (breastmilk or breast-milk or mothersmilk or mothers-milk or ((breast or human? or maternal or mother? or woman) adj2 milk)).ti,ab,kw.
7 (breastfe* or breast-fe* or (breast adj2 (feed* or fed))).ti,ab,kw.
8 (colostrum* or colostral* or colostrium or foremilk or fore-milk or hindmilk or hind-milk).ti,ab,kw.
9 (lactation or breast-secretion* or milk-release or ((breast or milk) adj2 (secretion* or release))).ti,ab,kw.
10 or/2-9
11 [Non-coding RNA concept]
12 nuclear RNA/
13 exp ribosome RNA/

14 exp transfer RNA/
15 exp untranslated RNA/
16 long untranslated RNA/
17 ribozyme/
18 exp small untranslated RNA/
19 piwi interacting rna/
20 short hairpin rna/
21 small cytoplasmic rna/
22 small interfering rna/
23 small nuclear rna/
24 small nucleolar rna/
25 spliced leader rna/
26 noncoding-ribonucleic-acid?.ti,ab,kw.
27 noncoding-RNA?.ti,ab,kw.
28 non-conding-ribonucleic-acid?.ti,ab,kw.
29 non-conding-RNA?.ti,ab,kw.
30 nonprotein-coding-ribonucleic-acid?.ti,ab,kw.
31 nonprotein-coding-RNA?.ti,ab,kw.
32 non-protein-coding-ribonucleic-acid?.ti,ab,kw.
33 non-protein-coding-RNA?.ti,ab,kw.
34 uncoding-ribonucleic-acid?.ti,ab,kw.
35 uncoding-RNA?.ti,ab,kw.
36 non-Messenger-ribonucleic-acid?.ti,ab,kw.
37 non-messenger-RNA?.ti,ab,kw.
38 junk-ribonucleic-acid?.ti,ab,kw.
39 junk-RNA?.ti,ab,kw.
40 garbage-ribonucleic-acid?.ti,ab,kw.
41 garbage-RNA?.ti,ab,kw.
42 untranslated-ribonucleic-acid?.ti,ab,kw.
43 untranslated-RNA?.ti,ab,kw.
44 nontranslated-RNA?.ti,ab,kw.
45 non-translated-ribonucleic-acid?.ti,ab,kw.
46 non-translated-RNA?.ti,ab,kw.
47 long-noncoding-ribonucleic-acid?.ti,ab,kw.
48 long-noncoding-RNA?.ti,ab,kw.
49 long-non-coding-RNA?.ti,ab,kw.
50 long-non-coding-ribonucleic-acid?.ti,ab,kw.
51 long-non-protein-coding-ribonucleic-acid?.ti,ab,kw.
52 long-non-protein-coding-RNA?.ti,ab,kw.
53 long-untranslated-ribonucleic-acid?.ti,ab,kw.
54 long-untranslated-RNA?.ti,ab,kw.
55 long-non-translated-ribonucleic-acid?.ti,ab,kw.
56 long-non-translated-RNA?.ti,ab,kw.
57 long-intergenic-non-protein-coding-ribonucleic-acid?.ti,ab,kw.
58 long-intergenic-non-protein-coding-RNA?.ti,ab,kw.
59 lincRNA?.ti,ab,kw.
60 linc-RNA?.ti,ab,kw.
61 lncRNA?.ti,ab,kw.
62 lnc-RNA?.ti,ab,kw.
63 ncRNA?.ti,ab,kw.
64 nc-RNA?.ti,ab,kw.

65 small-ribonucleic-acid?.ti,ab,kw.
66 small-RNA?.ti,ab,kw.
67 sRNA?.ti,ab,kw.
68 micro-ribonucleic-acid?.ti,ab,kw.
69 microRNA?.ti,ab,kw.
70 micro-RNA?.ti,ab,kw.
71 miRNA?.ti,ab,kw.
72 mi-RNA?.ti,ab,kw.
73 mirs.ti,ab,kw.
74 circulating-micro-ribonucleic-acid?.ti,ab,kw.
75 circulating-micro-RNA?.ti,ab,kw.
76 small-intefering-ribonucleic-acid?.ti,ab,kw.
77 small-intefering-RNA?.ti,ab,kw.
78 short-interfering-ribonucleic-acid?.ti,ab,kw.
79 short-interfering-RNA?.ti,ab,kw.
80 silencing-ribonucleic-acid?.ti,ab,kw.
81 silencing-RNA?.ti,ab,kw.
82 siRNA?.ti,ab,kw.
83 si-RNA?.ti,ab,kw.
84 trans-actins-siRNA?.ti,ab,kw.
85 tasiRNA?.ti,ab,kw.
86 tasi-RNA?.ti,ab,kw.
87 repeat-associated-ribonucleic-acid?.ti,ab,kw.
88 repeat-associated-RNA?.ti,ab,kw.
89 rasi-RNA?.ti,ab,kw.
90 rasiRNA?.ti,ab,kw.
91 piwi-interacting-ribonucleic-acid?.ti,ab,kw.
92 piwi-interacting-RNA?.ti,ab,kw.
93 piRNA?.ti,ab,kw.
94 pi-RNA?.ti,ab,kw.
95 short-haripin-ribonucleic-acid?.ti,ab,kw.
96 short-haripin-RNA?.ti,ab,kw.
97 small-hairpin-ribonucleic-acid?.ti,ab,kw.
98 small-hairpin-RNA?.ti,ab,kw.
99 sh-RNA?.ti,ab,kw.
100 shRNA?.ti,ab,kw.
101 small-scan-ribonucleic-acid?.ti,ab,kw.
102 small-scan-RNA?.ti,ab,kw.
103 scn-RNA?.ti,ab,kw.
104 scnRNA?.ti,ab,kw.
105 small-nucleolar-ribonucleic-acid?.ti,ab,kw.
106 small-nucleolar-RNA?.ti,ab,kw.
107 snoRNA?.ti,ab,kw.
108 sno-RNA?.ti,ab,kw.
109 small-nuclear-ribonucleic-acid?.ti,ab,kw.
110 small-nuclear-RNA?.ti,ab,kw.
111 snRNA?.ti,ab,kw.
112 sn-RNA?.ti,ab,kw.
113 small-cajal-body-specific-ribonucleic-acid?.ti,ab,kw.

114 small-cajal-body-specific-RNA?.ti,ab,kw.
115 scaRNA?.ti,ab,kw.
116 sca-RNA?.ti,ab,kw.
117 extracellular-ribonucleic-acid?.ti,ab,kw.
118 extracellular-RNA?.ti,ab,kw.
119 exosomal-ribonucleic-acid?.ti,ab,kw.
120 exosomal-RNA?.ti,ab,kw.
121 ex-RNA?.ti,ab,kw.
122 transfer-ribonucleic-acid?.ti,ab,kw.
123 transfer-RNA?.ti,ab,kw.
124 soluble-ribonucleic-acid?.ti,ab,kw.
125 soluble-RNA?.ti,ab,kw.
126 tRNA?.ti,ab,kw.
127 t-RNA?.ti,ab,kw.
128 tRF?.ti,ab,kw.
129 tRNA-derived-small-ribonucleic-acid?.ti,ab,kw.
130 tRNA-derived-small-RNA?.ti,ab,kw.
131 t-RNA-derived-small-RNA?.ti,ab,kw.
132 tsRNA?.ti,ab,kw.
133 ts-RNA?.ti,ab,kw.
134 ribosomal-ribonucleic-acid?.ti,ab,kw.
135 ribosomal-RNA?.ti,ab,kw.
136 ribosome-ribonucleic-acid?.ti,ab,kw.
137 ribosome-RNA?.ti,ab,kw.
138 rRNA?.ti,ab,kw.
139 r-RNA?.ti,ab,kw.
140 circular-ribonucleic-acid?.ti,ab,kw.
141 circular-RNA?.ti,ab,kw.
142 circRNA?.ti,ab,kw.
143 circ-RNA?.ti,ab,kw.
144 or/12-143
145 9 and 144

1.4 Web of Science

10 #9 AND #8

Indexes=SCI-EXPANDED, SSCI, A&HCI, CPCI-S, CPCI-SSH, ESCI Timespan=All years

9 TS=("noncoding-ribonucleic-acid?" or "noncoding-RNA?" or "non-conding-ribonucleic-acid?" or "non-conding-RNA?" OR "nonprotein-coding-ribonucleic-acid?" or "nonprotein-coding-RNA?" or "non-protein-coding-ribonucleic-acid?" or "non-protein-coding-RNA?" or "uncoding-ribonucleic-acid?" or "uncoding-RNA?" or "non-Messenger-ribonucleic-acid?" or "non-messenger-RNA?" or "junk-ribonucleic-acid?" or "junk-RNA?" or "garbage-ribonucleic-acid?" or "garbage-RNA?" or "untranslated-ribonucleic-acid?" or "untranslated-RNA?" or "nontranslated-RNA?" or "non-translated-ribonucleic-acid?" or "non-translated-RNA?" or "long-noncoding-ribonucleic-acid?" or "long-noncoding-RNA?" or "long-non-coding-RNA?" or "long-non-coding-ribonucleic-acid?" or "long-non-protein-coding-ribonucleic-acid?" or "long-non-protein-coding-RNA?" or "long-untranslated-ribonucleic-acid?" or "long-untranslated-RNA?" or "long-non-translated-ribonucleic-acid?" or "long-non-translated-RNA?" or "long-intergenic-non-protein-coding-ribonucleic-acid?" or "long-intergenic-non-protein-coding-RNA?" or "lincRNA?" or "linc-RNA?" or "lncRNA?" or "lnc-RNA?" or "ncRNA?" or "nc-RNA?" or "small-ribonucleic-acid?" or "small-RNA?" or "sRNA?" or "micro-ribonucleic-acid?" or "microRNA?" or "micro-RNA?" or "miRNA?" or "mi-RNA?" or "mirs" or "circulating-micro-ribonucleic-acid?" or "circulating-micro-RNA?" or "small-intefering-ribonucleic-acid?" or "small-intefering-RNA?" or "short-interfering-ribonucleic-acid?" or "short-interfering-RNA?" or "silencing-ribonucleic-acid?" or "silencing-RNA?" or "siRNA?" or "si-RNA?" or "trans-actins-siRNA?" or "tasiRNA?" or "tasi-

RNA?" or "repeat-associated-ribonucleic-acid?" or "repeat-associated-RNA?" or "rasi-RNA?" or "rasiRNA?" or "piwi-interacting-ribonucleic-acid?" or "piwi-interacting-RNA?" or "piRNA?" or "pi-RNA?.ti,ab,kw. short-haripin-ribonucleic-acid?" or "short-haripin-RNA?" or "small-hairpin-ribonucleic-acid?" or "small-hairpin-RNA?" or "sh-RNA?" or "shRNA?" or "small-scan-ribonucleic-acid?" or "small-scan-RNA?" or "scn-RNA?" or "scnRNA?" or "small-nucleolar-ribonucleic-acid?" or "small-nucleolar-RNA?" or "snoRNA?" or "sno-RNA?" or "small-nuclear-ribonucleic-acid?" or "small-nuclear-RNA?" or "snRNA?" or "sn-RNA?" or "small-cajal-body-specific-ribonucleic-acid?" or "small-cajal-body-specific-RNA?" or "scaRNA?" or "sca-RNA?" or "extracellular-ribonucleic-acid?" or "extracellular-RNA?" or "exosomal-ribonucleic-acid?" or "exosomal-RNA?" or "ex-RNA?" or "transfer-ribonucleic-acid?" or "transfer-RNA?" or "soluble-ribonucleic-acid?" or "soluble-RNA?" or "tRNA?" or "t-RNA?" or "tRF?" or "tRNA-derived-small-ribonucleic-acid?" or "tRNA-derived-small-RNA?" or "t-RNA-derived-small-RNA?" or "tsRNA?" or "ts-RNA?" or "ribosomal-ribonucleic-acid?" or "ribosomal-RNA?" or "ribosome-ribonucleic-acid?" or "ribosome-RNA?" or "rRNA?" or "r-RNA?" or "circular-ribonucleic-acid?" or "circular-RNA?" or "circRNA?" or "circ-RNA?")

Indexes=SCI-EXPANDED, SSCI, A&HCI, CPCI-S, CPCI-SSH, ESCI Timespan=All years

8 #7 OR #6 OR #5 OR #4 OR #3 OR #2 OR #1

Indexes=SCI-EXPANDED, SSCI, A&HCI, CPCI-S, CPCI-SSH, ESCI Timespan=All years

7 **TOPIC:** (("breast" or "milk") NEAR/2 ("secretion*" or "release"))

Indexes=SCI-EXPANDED, SSCI, A&HCI, CPCI-S, CPCI-SSH, ESCI Timespan=All years

6 **TOPIC:** ("lactation" or "breast-secretion*" or "milk-release")

Indexes=SCI-EXPANDED, SSCI, A&HCI, CPCI-S, CPCI-SSH, ESCI Timespan=All years

5 **TOPIC:** ("colostrum*" or "colostral*" or "colostrium" or "foremilk" or "fore-milk" or "hindmilk" or "hind-milk")

Indexes=SCI-EXPANDED, SSCI, A&HCI, CPCI-S, CPCI-SSH, ESCI Timespan=All years

4 **TOPIC:** ("breast" NEAR/2 ("feed*" or "fed"))

Indexes=SCI-EXPANDED, SSCI, A&HCI, CPCI-S, CPCI-SSH, ESCI Timespan=All years

3 **TOPIC:** ("breastfe*" or "breast-fe*")

Indexes=SCI-EXPANDED, SSCI, A&HCI, CPCI-S, CPCI-SSH, ESCI Timespan=All years

2 **TOPIC:** (("breast" or "human?" or "maternal" or "mother?" or "woman") NEAR/2 "milk")

Indexes=SCI-EXPANDED, SSCI, A&HCI, CPCI-S, CPCI-SSH, ESCI Timespan=All years

1 **TOPIC:** ("breastmilk" or "breast-milk" or "mothersmilk" or "mothers-milk")

Indexes=SCI-EXPANDED, SSCI, A&HCI, CPCI-S, CPCI-SSH, ESCI Timespan=All years

2 Approach to assessment of quality

To our knowledge, there are not quality or bias assessment checklists specifically developed for miRNA analyses. We have therefore constructed a checklist incorporating questions from both NICE ("Quality appraisal checklists - quantitative studies reporting correlations and associations") and derived from an article Han et al. entitled "A checklist is associated with increased quality of reporting preclinical biomedical research: A systematic review".

Specifically, we extracted information on:

Study design

- clarity of the research question(s) and aim(s)
- sample characteristics and clarity of inclusion and exclusion criteria
- clarity of details regarding sample collection (lactational stage, time, method), number of samples and samples storage
- risk of misclassification of exposure or outcome (clarity of criteria for these groups)

Laboratory analyses

- clear description of laboratory methods (sample processing, RNA extraction, RNA quantification)
- risk of bias due to differences in processing/analysing samples being compared;
- the use of technical and biological replicates

Bioinformatics pipeline, statistics and predictions

- adequate description of data processing (particularly for RNAseq)
- use of power calculations; sufficiently described analytical methods for breast milk samples;
- adequately described and relevant statistical methods;
- normalisation of the data;
- multiple hypothesis testing addressed
- adequate description of databases, software and experimental material

Results

- clarity of results presentation in figures and texts;
- Presentation of effect estimates (graphically or numerically);
- open access data available (for studies which employ sequencing);
- inclusion of supplementary material if relevant

Disagreements between the reviewers were resolved through discussion.

Assessments will be performed for each study by two reviewers and were rated from 0 to 3 where:

3 (++)	Indicates that the particular aspect has been designed or conducted in such a way as to minimise the risk of bias
2 (+)	Indicates that either it is not clear from the way the study is reported, or that the study may not have addressed all potential sources of bias for that particular aspect
1 (-)	Should be reserved for those aspects of the study design in which significant sources of bias may persist
0 (NR)	Should be reserved for those aspect in which the study under review fails to report how they have (or might have) been considered
NA	Should be reserved for those study design aspects that are not applicable given the study design under review.

3 Supplementary results

3.1 Table S1: Description of read proportions in RNAseq studies

Author	Fraction (n)	No. of clean reads	Proportion miRNA (%)	Proportion of miRNAs reads due to top 10 miRNAs	Other RNAs described	Fresh/ frozen	Library Preparation	Library size selection	Illumina platform
Alsaweed, 2016[1]	Cell (n = 20)	268 681 616	65	73.7	Also report: piRNA; rRNA; scRNA; snRNA; snoRNA; tRNA; exact proportions unclear.	Fresh	Solexa	NR[a]	HiSeq 2000
Alsaweed, 2016[2]	Cell (n = 30)<br>Lipid (n = 15)	350 181 081<br>195 474 461	58<br>40	72.4<br>75.7	The following RNAs are mentioned, but no overall proportions provided: rRNA; scRNA; snRNA; snoRNA; tRNA; srpRNA	Fresh	Solexa	NR[a]	HiSeq 2000
Carney, 2017[3]	Lipid (n = 67)<br>Skim (n = 67)	NR<br>NR	NR<br>NR	~60.0[b]<br>NR	None reported	Frozen	NEXTFlex	NR	HiSeq 2000
Golan-Gerstl, 2017[4]	Skim milk (n = unclear)[c]<br>Lipid (n = unclear)[c]	6472862<br>3785876	22<br>11	72.9<br>65.3	None reported	Frozen	NEBNext	NR	NextSeq 500
Kahn, 2018[5]	EVs (preterm, n = 10)<br>EVs (term,n = 10)	NR<br>NR	NR<br>NR	Unclear[d]	None reported	Frozen	TruSeq	Blue Pippen 3 % agarose cassette; size NR	HiSeq 2500
Leiferman, 2019[6]	EVs (n = 3)	NR	NR	71.0	None reported	Fresh	NEXTFlex	NR	HiSeq 2500
Liao, 2017[7]	EVs (n = 12)	NR	42	63.8[d]	None reported	Frozen	NEXTFlex	NR	HiSeq 2500
Munch, 2013[8]	Lipid (n = 6)	124 110 646	25	81.1	None reported	Frozen	NR	NR	Genome Analyzer (1G)
Rubio, 2018[9]	Skim milk (n = 10)	125 151 530	36	46.8	Also reported short reads from tRNAs (30.9 %); piRNA (8.4 %); rRNA, snoRNA, snRNA, lncRNA, VTRNA (all <1%)	Centrifuged before freezing[e]	NEBNext	Acrylamide gel; size NR	HiSeq 2000
Simpson, 2015[10]	EVs (n = 54)	1 314 187 263	9	63.0	Also reported short reads from tRNAs (35.5 %); rRNAs (40.3 %) and "other" RNAs.	Frozen	ScriptMiner	Acrylamide gel; selected 65-82 nt (equal to reads of 11-28 nt)	HiSeq 2000
Smycsyncska, 2020[11]	Whole milk (n = 3)<br>EVs (n = 3)	16 649 388<br>21 535 212	5<br>16	64.3<br>69.0	High proportion described as "unknown function" or unmapped	Frozen	QIASeq	Unclear	NextSeq 500
van Herwijnen, 2018[12]	EVs (n = 4, pooled)	1 680 013	0.6	34.6	Also reported reads from rRNAs (3.51 %); otherwise no description of origin of the other ~96 % of reads.	Fresh	NEBNext	146-400 nt (implied that this is equal to reads of 20-124 nt)	HiSeq 2000
Zhou, 2012[13]	EVs (n = 4)	~83.52 M reads	NR	62.3	None reported	Frozen	NR	NR	Genome Analyzer II

[a]Prior to library preparation, size fractionation is described to select small RNAs of 18-30 nt; [b]This is reported as an approximation because the read counts in the supplementary files used to calculate this proportion are rounded to the nearest 100 or 1000; [c]Unclear how many human samples underwent sequencing; [d]In Kahn et al and Liao et al the percentage of reads associated with the top 15 miRNA appears to be calculated based on logarithmic transformed normalized counts and reported as 11-12 %, for Liao et it was possible re-calculate and present the percentage of reads associated with the top 10 miRNA based on raw read counts presented in supplementary files as the values; [e]Samples were centrifuged whilst fresh and the skim milk fraction was stored frozen for later analysis. Kupsco et al[14] not included in this summary since HTG EdgeSeq technology targets only miRNAs.

3.2 Table S2: Top 10 novel miRNA candidates described in human milk

Alsaweed 2016[1]				Alsaweed 2016[2]				Munch 2013[8]			
**Assigned name**	**Sequence**	**Total reads**	**No. of samples**	**Assigned name**	**Sequence**	**Total reads**	**No. of samples**	**Assigned name**	**Sequence**	**Total reads**	**No. of samples**
novel_mir_7	**TCCATATCCCAACCTGTCAGAGT***	3890	13 / 20	novel_mir_189	GCCTGTCTGAGCGTCGCT	751632	13 / 45	Novel-miR-102	TCCATATCCCAACCTGTCAGAGT	2201	5 / 6
novel_mir_299	ACTAGGATTGTGCTTCCCTGG	942	19 / 20	novel_mir_2	ATGTTGGATCAGGACATCC	18054	20 / 45	Novel-miR-79	TTTTTTGCTGGAACATTTCTGG	1639	5 / 6
novel_mir_367	TGCACGCGACCATAGAGCCT	804	17 / 20	novel_mir_112	GACCTCGCCGTCCCGCCCGCC	5608	8 / 45	Novel-miR-85	ATTAGGTAGTGGCAGTGGAAC	205	4 / 6
novel_mir_39	TCTGGCATGGCCTTGGGCACT	789	20 / 20	novel_mir_4	TCCATATCCCAACCTGTCAGAGT	5581	27 / 45	Novel-miR-114	GTGCGTGGTGGCTCGAGGCGGG	117	4 / 6
novel_mir_115	CAACCCCGGGCTGATCACTACT	760	17 / 20	novel_mir_5	AATGTGGCTTAGAACATG	3495	27 / 45	Novel-miR-67	TTGAGGGGAGAATGAGGTGGAGA	94	3 / 6
novel_mir_476	**TCCATATCCCAACCTGTCAGAG***	658	4 / 20	novel_mir_392	GCGCGCCCCCGCCCCGGC	1520	14 / 45	Novel-miR-27	TCTCACCTGGCATAAGCAATT	82	3 / 6
novel_mir_90	ACTGGCAAAAGGGTTTAGAACT	586	17 / 20	novel_mir_472	TAGACGGGCTCACATCACC	1288	10 / 45	Novel-miR-109	ACGCGATTTGTAGCACAGACA	63	3 / 6
novel_mir_41	GGGCGTTGCTGGGCGTTGCT	341	12 / 20	novel_mir_1905	TTAGGTCAAGGTGTAGCC	1242	2 / 45	Novel-miR-118.2	TTGAACTCGAGTTGGAAGAGGCG	54	2 / 6
novel_mir_269	TACTTGACCTTGACTCTCCCT	322	15 / 20	novel_mir_471	GACCTCGCCGTCCCGCCCGCCG	1200	2 / 45	Novel-miR-123	AGCAAAGCAAAGCTCAGTTGGA	53	3 / 6
novel_mir_161	TCTGAGACTAGAGCAAAGCCCT	295	9 / 20	novel_mir_2797	GTCGGGGCGGCGGCGGCGGCG	1104	2 / 45	Novel-miR-44	TCAGCTACTACCTCTATTAGGA	36	3 / 6

*The sequences annotated as novel-mir-7 and novel-mir-476 are examples of overlapping sequences which are assigned unique names but are derived from the same genomic location and differ by only one nucleotide at either the 3’ or 5’ end of the sequence. Many such reads are found with unique novel miRNA candidate IDs the supplementary files associated with the article by Alsaweed and coauthors[1,2].

3.3 Table S3: Quality assessment of included studies

	Alsaweed 2015	Alsaweed 2016	Alsaweed 2016 (Feb)	Alsaweed 2016 (Apr)	Bozack 2020	Carney 2017	Floris 2015	Golan-Gerstl 2017	Kahn 2018	Karlsson 2016	Kosaka 2010	Kupsco 2021	Leiferman 2019	Liao 2017	Munch 2013	Na 2015	Perri 2018	Qin 2017	Rubio 2018	Shah 2021	Shiff 2019	Simpson 2015	Smycsyncska 2020	van Herwijnen 2018	Weber 2010	Wu 2020	Xi 2016	Zamanillo 2019	Zhou 2012	Zhou 2021
Quality and transparency of study: mean score of (24) parameters assessed below																														
**1. Study design**																														
1.1 Are the research question(s) and aim(s) clearly described?	3	3	3	3	3	3	2	3	3	3	2	3	3	3	2	3	3	3	3	3	3	3	3	2	3	3	2	3	3	3
1.2 Were the following aspects of the study design and procedure described clearly?:																														
1.2.1 Inclusion and exclusion criteria	3	3	3	3	3	2	2	2	3	2	2	3	2	2	2	1	3	2	3	3	3	3	3	1	1	1	3	2	3	2
1.2.2 Sample collection (date, time, procedure)	2	3	3	2	3	2	1	1	2	3	2	2	2	2	3	2	2	2	2	2	2	2	2	1	0	2	3	3	2	2
1.2.3 Number of samples	2	2	3	3	3	3	3	2	3	3	3	3	2	3	3	3	3	3	3	3	3	3	3	3	3	3	3	3	3	3
1.2.4 Sample storage	3	3	3	3	3	2	3	2	2	2	2	2	2	2	2	2	3	2	2	2	3	3	3	2	0	3	3	2	2	3
1.3 Is there a risk of misclassification of exposure and/or outcome :																														
1.3.1 Exposure(s), if maternal/infant characteristics included	NA	3	NA	3	3	3	3	NA	3	NA	NA	3	NA	NA	3	NA	3	NA	NA	3	3	3	NA	NA	NA	NA	3	3	NA	3
1.3.2 Outcome(s), if infant outcomes included	NA	NA	NA	NA	NA	NA	NA	NA	NA	NA	NA	NA	NA	NA	NA	NA	NA	NA	NA	3	NA	3	NA	NA	NA	NA	NA	3	NA	NA
Mean scoring (excl. NA):	2.6	2.8	3.0	2.8	3.0	2.5	2.3	2.0	2.7	2.6	2.2	2.7	2.2	2.4	2.5	2.2	2.8	2.4	2.6	2.7	2.8	2.9	2.8	1.8	1.4	2.4	2.8	2.7	2.6	2.7
**2 Laboratory analyses**																														
2.1 Were the following methodological aspects described clearly?:																														
2.1.1 Sample processing	3	3	3	3	3	3	3	3	3	3	3	3	3	3	3	1	2	3	3	3	3	3	3	3	2	2	3	3	3	3
2.1.2 RNA extraction - adequate details	3	3	3	3	3	3	3	3	3	3	3	2	2	3	3	3	3	2	3	3	3	3	3	3	2	2	3	3	3	2
2.1.3 RNA quantification - adequate details	2	3	3	3	3	3	3	2	2	3	3	2	3	3	3	3	3	3	3	3	3	3	3	3	3	2	3	3	3	3
2.2 Were the same methods used for all samples being compared?	1	3	1	1	3	2	3	3	3	3	3	3	NA	3	2	3	3	3	3	3	3	3	3	3	3	3	3	3	3	3
2.3 Were technical replicates used?	0	0	2	0	0	0	3	0	0	0	2	0	0	0	0	0	3	0	0	3	0	0	0	0	0	3	3	0	2	0
2.4 Were biological replicates used?	3	3	3	3	3	3	3	3	3	3	3	3	2	3	3	3	3	3	3	3	3	3	2	3	1	3	3	3	3	3
Mean scoring (excl. NA):	2.0	2.5	2.5	2.2	2.5	2.3	3.0	2.3	2.3	2.5	2.8	2.2	2.0	2.5	2.3	2.2	2.8	2.3	2.5	3.0	2.5	2.5	2.3	2.5	1.8	2.5	3.0	2.5	2.8	2.3
**3 Bioinformatics pipeline, statistics and predictions (I'VE LEFT THIS SECTION FOR U, MEL)**																														
3.1 Is data processing adequately described?	3	2	3	2	3	3	3	1	0	3	0	3	2	3	3	0	3	3	3	2	3	3	3	3	2	2	3	2	3	3
3.2 Were any sample size calculations reported?	1	1	1	1	0	3	0	0	0	0	0	0	0	0	0	2	3	0	0	0	0	0	0	0	NA	0	0	0	0	0
3.3 Are the statistical methods adequately described?	3	1	2	1	3	3	3	1	1	3	0	3	2	3	2	0	3	3	3	3	0	3	3	NA	NA	2	3	3	NA	2
3.4 Are the statistical methods relevant?	3	2	3	2	2	2	3	1	1	3	0	3	2	3	3	2	3	3	3	3	0	3	3	NA	NA	3	3	2	NA	3
3.5 Was there data normalisation prior to statistical analysis?	NA	1	3	1	3	3	NA	1	1	NA	NA	3	3	1	3	NA	NA	3	3	2	2	3	3	1	1	3	3	3	3	3
3.6 Was multiple hypothesis testing addressed?	1	0	0	0	0	3	NA	0	0	0	0	3	3	3	3	NA	NA	NA	3	0	0	3	2	NA	NA	0	NA	0	NA	1
3.7 Is there adequate description of databases, software and experimental material?	NA	3	3	3	3	3	NA	1	0	NA	0/NA	3	NA	3	3	NA	NA	NA	3	NA	NA	3	3	3	NA	3	NA	NA	3	3
Mean scoring (excl. NA):	2.2	1.4	2.1	1.4	2.0	2.9	2.3	0.7	0.4	1.8	0.0	2.6	2.0	2.3	2.4	1.0	3.0	2.4	2.6	1.7	0.8	2.6	2.4	1.8	1.5	1.9	2.4	1.7	2.3	2.1
**4 Results**																														
4.1 Are the results presented clearly in the text, figures and tables?	3	2	3	2	3	2	3	3	2	3	2	3	3	3	3	3	3	2	3	3	3	3	3	2	3	3	3	3	3	3
4.2 Are effect estimates presented (either graphically or numerically)?	NA	2	2	2	3	3	3	2	2	3	3	3	3	3	3	3	3	2	3	3	3	3	3	NA	NA	3	3	3	2	2
4.3 Is the raw data openly available (for studies which employ sequencing)?	NA	3	3	3	0	0	NA	0	0	NA	NA	3	3	0	0	NA	NA	NA	3	0	0	0	3	3	0	0	NA	0	3	0
4.4 Inclusion of supplementary material if relevant	NA	3	3	3	2	3	3	2	3	3	0	3	3	3	3	NA	NA	0	3	3	NA	3	3	3	NA	0	NA	3	3	3
Mean scoring (excl. NA):	3.0	2.5	2.8	2.5	2.0	2.0	3.0	1.8	1.8	3.0	1.7	3.0	3.0	2.3	2.3	3.0	3.0	1.3	3.0	2.3	2.0	2.3	3.0	2.7	1.5	1.5	3.0	2.3	2.8	2.0
Mean of all items scored (excl. all NA):	2.5	2.3	2.6	2.2	2.4	2.4	2.6	1.7	1.8	2.5	1.7	2.6	2.3	2.4	2.4	2.1	2.9	2.1	2.7	2.4	2.0	2.5	2.6	2.2	1.6	2.1	2.8	2.3	2.6	2.3

(++)	3	Indicates that for that particular aspect of study design, the study has been designed or conducted in such a way as to minimize the risk of bias
(+)	2	Indicates that either it is not clear from the way the study is reported, or that the study may not have addressed all potential sources of bias for that particular aspect of study design.
(-)	1	Should be reserved for those aspects of the study design in which significant sources of bias may persist.
NR	0	Should be reserved for those aspects in which the study under review fails to report how they have (or might have) been considered.
NA	NA	Should be reserved for those study design aspects that are not applicable given the study design under review.

3.4 Table S4: Statistical approach in studies employing targeted PCR only

Author	Comparison	Statistical / bioinformatic method of comparison	Adj. for multiple comparisons
Alsaweed (2015)[15]	Milk fraction	Linear regression or linear mixed effects model used to compare differences efficiency in extracting total RNA and miRNA, purity of RNA (260/280 ratio), ratio of miRNA to small RNA between kits and milk fractions. The use of ANOVA is also described, although where these are presented.	No (ANOVA accompanied by Tukey's HSD)
Floris (2015)[16]	Milk fraction; Diurnal fluctuations; Lactational stage; Milk storage	Comparisons between miRNA Cq data in whole milk, lipids and skim milk were performed using one-way ANOVA (with Tukey's post hoc multiple comparison test). One-way ANOVA (with Tukey's post hoc multiple comparison test) used to assess miRNA variations throughout the 24 hours. Geometric mean of let-7g/d or let-/g/d/miR-146b used as normalization factors. Unclear how difference across lactational stages and milk storage conditions were assessed statistically.	Yes, Tukey HSD
Karlsson (2016)[17]	None	Expression levels for the 87 lncRNAs correlated with one another using Spearman's Rank Correlation test. Otherwise no associations investigated with maternal or infant characteristics.	NA
Na (2015)[18]	[Lactational stage]	Not described. Appears to have only collected colostrum from humans, and analyses of lactational stages was performed on goat milk.	
Perri (2018)[19]	Methodology; Lactational stage	One-way ANOVA and Newman-Keuls Multiple Comparison Test used to assess differences in mean miR-21, miR-181a, miR-150, miR-223 abundance between colostrum and mature milk.	No
Qin (2017)[20]	Milk fraction	Correlations or differences between milk fractions was not appear to have been formally tested. Pairwise correlation of relative expression levels between miRNAs within each milk fractions assessed using Pearson's correlation coefficient, however data from these analyses is not presented in the article.	No
Shah (2021)[21]	Maternal weight; Lactational stage; Infant growth and body composition	Association with maternal weight and lactational stage tested with either unpaired t-test or Mann-Whitney U-test. The associations with lactational stages also tested using ANCOVA and adjusting for maternal weight status (overweight/obese or normal weight). Associations with infant growth and body composition assessed with linear regression, including "… 1- and 3-month milk miRNA fold change as independent variables… [and controlling] for gestational age, birth weight and infant sex". Interpretation of these coefficients are somewhat unclear since an increase in fold change refers to an increase in the relative expression levels of miRNA of the overweight/obese group compared to the normal weight group.	No
Shiff (2020)[22]	Lactational stage; Term/preterm	Not described	No
Xi (2016)[23]	Lactational stage; Maternal age, weight, BMI; gestational length; Infant sex	Log transformed data. Associations with lactational stage tested with paired t-test. Pearson's or Spearman's correlation used to assess associations with maternal age, weight, BMI, gestational length. ANOVA used to assess associations between miRNA levels and infant sex & gestational metabolic disease (gestational hypertensive disease and gestational diabetes mellitus). ANCOVA used to control for maternal BMI when assessing association between miRNA levels and gestational metabolic complications (data for this analysis not presented).	No
Zamanillo (2019)[24]	Lactational stage; Maternal BMI, milk leptin/adiponectin; Infant health	Repeated measures ANOVA to assess impact of time and maternal BMI Spearman's correlation for association between miRNA and milk leptin/adiponectin, and with infant BMI at 24 months	No

Tukey's HSD: Tukey's honestly significant difference test

3.5 Table S5: Statistical approach in studies employing RNAseq or TaqMan OpenArray

Author	Quantification method	Comparison	Statistical / bioinformatic method of comparison	Adj. for multiple comparisons
Alsaweed (2016a)[1]	RNAseq & qPCR	Fore-/hindmilk	**RNAseq:** DEGseq to find p-values for fold expression change between fore- and hindmilk samples **PCR:** hsa-let-7f-5p, hsa-miR-181a-5p, hsa-miR-148a-3p, hsa-miR-22-3p, and hsa-miR-182-5p compared in fore- and hindmilk samples using linear mixed effects models. The 4 most highly expressed novel RNA were also assessed using PCR.	Unclear
Alsaweed (2016b)[25]	TaqMan OpenArray	Milk fraction	Comparison of milk fractions conducted using linear models for microarray analysis (limma) via HTqPCR package in R.	Appears to be Tukey's HSD
Alsaweed (2016c)[2]	RNAseq	Lactational stage	DEGseq to find p-values for fold expression change between lactational stage and between cell and lipid fraction	Unclear
Bozack (2020)[26]	TaqMan OpenArray	Maternal BMI, education, smoking, age, lifetime stress and negative life events; infant sex; race/ethnicity	Mann-Whitney and Kruskal-Wallis tests for associations between maternal-infant characteristics and number of EV-miRNAs; Spearman correlation to assess associations between maternal stress and number of EV-miRNAs. Logistic regression used to assess association between miRNA detection and maternal stress (adjusted for infant sex, maternal race, maternal education, stage of lactation). Robust linear regression (*rlm* function in R) used to assess associations between miRNA expression and infant sex, maternal characteristics (age, race, education, pre-pregnancy obesity, tobacco smoke exposure during pregnancy), lactational stage, and measures of maternal stress.	No
Carney (2017)[3]	RNAseq	Lactational stage; Fore-/hindmilk; term/preterm	**Differential expression:** GSA analysis used to identify individual miRNAs with significant differences between tMBM and pMBM fractions. Fold change in miRNA levels between groups was reported as log2 values. Also describes that Kruskal‑Wallis test was used to examine differences in individual miRNAs across lipid fractions of tMBM, pMBM, and tColostrum. **Other analyses:** Two-dimensional PLS-DA to assess total miRNA profile. Hierarchical clustering with Pearson distance measure for top 20 miRNAs on Kruskal‑Wallis testing. Pearson correlation coefficient to assess relationship between maternal/infant medical and demographic characteristics and MBM miRNAs.	FDR for GSA analysis<br>None reported for Kruskal-Wallis test, or correlation
Golan-Gerstl (2017)[4]	RNAseq & qPCR	Milk fraction	No formal statistical comparisons appear to be undertaken for either RNAseq or qPCR data.	NA
Khan (2018)[5]	RNAseq	Term/preterm	Paired two-tailed Student's t-test to assess effect of *in vitro* digestion.	No
Kosaka (2010)[27]	MA & qPCR	Lactational stage; freeze-thaw cycles	Not described	No
Kupsco (2021)[14]	HTG EdgeSeq technology	Lactational stage; Maternal BMI, parity, smoking.	Unsupervised hierarchical clustering to identify and investigate clusters of miRNAs; Pearson's correlation to explore correlations between miRNAs across the top three clusters; Generalised linear model with negative binomial link function used to estimate associations between miRNAs and lactational stage, maternal BMI, parity and smoking. Each miRNA modelled individually with all maternal factors included simultaneously, together with maternal age. Normalisation performed using DESeq2, but unclear if the generalized linear models were also fitted using DESeq2.	Yes, FDR
Leiferman (2019)[6]	RNAseq & qPCR	[comparison to infant formula]	No formal comparison of sequencing data; qPCR used to compare human milk to infant formulas.	NA
Liao (2017)[7]	RNAseq	Lactational stage	Student's t-test was used to test the difference in microRNA abundance fold-change values between the undigested and digested groups, and the mean of the microRNA proportion in small RNAs before and after in vitro digestion.	No
Munch (2013)[8]	RNAseq & qPCR	Methodology; Milk fraction; Maternal characteristics	**RNA seq:** limma used in differential analysis comparing experimental maternal diet groups **PCR:** All data and statistical analysis was performed using Excel (Microsoft) and ANOVA (Partek). A p-value of ,0.05 was considered statistically significant.	

Table S5 continued:

Author	Quantification method	Comparison	Statistical / bioinformatic method of comparison	Adj. for multiple comparisons
Rubio (2018)[9]	RNAseq	Milk vs plasma	No comparison of breast milk miRNA based on methodology, maternal or infant characteristics<br>**Differential expression:** DESeq2 used for normalization and differential expression in comparison of milk to plasma<br>**Other analyses:** Principal Component Analysis (PCA) and dendrograms used to assess the impact of main technical and demographic/biological variables (R package: FactoMineR).	Yes, FDR
Simpson (2015)[10]	RNAseq	Maternal probiotic supplementation; Infant eczema	**Differential expression:** limma in comparison of miRNA between probiotic and placebo group and development of eczema	Yes, FDR
Smycsyncska (2020)[11]	RNAseq	Methodology; Milk fraction	**Differential expression:** DESeq2 used to compare miRNAs in raw and processed milk	Yes, FDR
van Herwijnen (2018)[12]	RNAseq	Comparison with other mammals	N/A. Not comparison as the human sample consisted of samples pooled from four individuals.	N/A
Weber (2010)[28]	Human miScript Assay	Lactational stage	No formal statistical comparisons employed to compare miRNA expression level in breast milk compared to colostrum. Data from this study not described in narrative summary because of insufficient details regarding timing of sample collection and insufficient numbers (n = 5 for mature milk; n = 1 for colostrum)	NA
Wu (2020)[29]	qPCR & MA	Lactational stage	**Differential expression:** edgeR to compare colostrum and mature milk. **qPCR:** Students two-tail t-test for comparison between colostrum and mature milk. Also describe use of ANOVA for comparison of more than two groups, however unclear where this has been employed.	No
Zhou (2012)[13]	RNAseq & qPCR	None	N/A	N/A
Zhou (2021)[30]	Arraystar Human circRNA Array v2	Gestational age	Limma used for hierarchical clustering to identify circRNA clusters and volcanic plots to find differentially expressed circRNAs between term and preterm milk samples.	No

1. Alsaweed M, et al. Human milk cells contain numerous miRNAs that may change with milk removal and regulate multiple physiological processes. *International Journal of Molecular Sciences* 2016; **17 (6) (no pagination)**(956).
2. Alsaweed M, et al. Human Milk Cells and Lipids Conserve Numerous Known and Novel miRNAs, Some of Which Are Differentially Expressed during Lactation. *PLoS ONE [Electronic Resource]* 2016; **11**(4): e0152610.
3. Carney MC, et al. Metabolism-related microRNAs in maternal breast milk are influenced by premature delivery. *Pediatric Research* 2017; **82**(2): 226-36.
4. Golan-Gerstl R, et al. Characterization and biological function of milk-derived miRNAs. *Molecular nutrition & food research* 2017; **61**(10): 10.
5. Kahn S, et al. Exosomal MicroRNAs in Milk from Mothers Delivering Preterm Infants Survive in Vitro Digestion and Are Taken Up by Human Intestinal Cells. *Molecular nutrition & food research* 2018; **62**(11): e1701050.
6. Leiferman A, et al. Storage of Extracellular Vesicles in Human Milk, and MicroRNA Profiles in Human Milk Exosomes and Infant Formulas. *Journal of Pediatric Gastroenterology & Nutrition* 2019; **69**(2): 235-8.
7. Liao YL, et al. Human milk exosomes and their microRNAs survive digestion in vitro and are taken up by human intestinal cells. *Molecular nutrition & food research* 2017; **61**(11): 11.
8. Munch EM, et al. Transcriptome profiling of microRNA by Next-Gen deep sequencing reveals known and novel miRNA species in the lipid fraction of human breast milk. *PLoS ONE [Electronic Resource]* 2013; **8**(2): e50564.
9. Rubio M, et al. Circulating miRNAs, isomiRs and small RNA clusters in human plasma and breast milk. *PLoS ONE [Electronic Resource]* 2018; **13**(3): e0193527.
10. Simpson MR, et al. Human Breast Milk miRNA, Maternal Probiotic Supplementation and Atopic Dermatitis in Offspring. *PLoS ONE [Electronic Resource]* 2015; **10**(12): e0143496.
11. Smyczynska U, et al. Impact of processing method on donated human breast milk microRNA content. *PLoS ONE [Electronic Resource]* 2020; **15**(7): e0236126.
12. van Herwijnen MJC, et al. Abundantly Present miRNAs in Milk-Derived Extracellular Vesicles Are Conserved Between Mammals. *Frontiers in Nutrition* 2018; **5**: 6.
13. Zhou Q, et al. Immune-related MicroRNAs are Abundant in Breast Milk Exosomes. *International Journal of Biological Sciences* 2012; **8**(1): 118-23.
14. Kupsco A, et al. Human milk extracellular vesicle miRNA expression and associations with maternal characteristics in a population-based cohort from the Faroe Islands. *Sci Rep* 2021; **11**(1): 5840.
15. Alsaweed M, et al. Human Milk MicroRNA and Total RNA Differ Depending on Milk Fractionation. *Journal of Cellular Biochemistry* 2015; **116**(10): 2397-407.
16. Floris I, et al. MiRNA Analysis by Quantitative PCR in Preterm Human Breast Milk Reveals Daily Fluctuations of hsa-miR-16-5p. *PLoS ONE [Electronic Resource]* 2015; **10**(10): e0140488.

17. Karlsson O, et al. Detection of long non-coding RNAs in human breastmilk extracellular vesicles: Implications for early child development. *Epigenetics* 2016; **11**(10): 721-9.
18. Na RS, et al. Expressional analysis of immune-related miRNAs in breast milk. *Genetics and Molecular Research* 2015; **14**(3): 11371-6.
19. Perri M, et al. Variation in Immune-Related microRNAs Profile in Human Milk Amongst Lactating Women. *MicroRNA* 2018; **7**(2): 107-14.
20. Qin WY, et al. Human Milk and Matched Serum Demonstrate Concentration of Select miRNAs. *Breastfeeding Medicine* 2017; **12**(1): 63-6.
21. Shah KB, et al. Human Milk Exosomal MicroRNA: Associations with Maternal Overweight/Obesity and Infant Body Composition at 1 Month of Life. *Nutrients* 2021; **13**(4).
22. Shiff YE, et al. MiRNA-320a is less expressed and miRNA-148a more expressed in preterm human milk compared to term human milk. *Journal of Functional Foods* 2019; **57**: 68-74.
23. Xi Y, et al. The levels of human milk microRNAs and their association with maternal weight characteristics. *European Journal of Clinical Nutrition* 2016; **70**(4): 445-9.
24. Zamanillo R, et al. Breast Milk Supply of MicroRNA Associated with Leptin and Adiponectin Is Affected by Maternal Overweight/Obesity and Influences Infancy BMI. *Nutrients* 2019; **11**(11): 28.
25. Alsaweed M, et al. Human milk miRNAs primarily originate from the mammary gland resulting in unique miRNA profiles of fractionated milk. *Scientific reports* 2016; **6**: 20680.
26. Bozack AK, et al. Associations between maternal lifetime stressors and negative events in pregnancy and breast milk-derived extracellular vesicle microRNAs in the programming of intergenerational stress mechanisms (PRISM) pregnancy cohort. *Epigenetics: Official Journal of the DNA Methylation Society* 2020: 1-16.
27. Kosaka N, et al. MicroRNA as a new immune-regulatory agent in breast milk. *Silence* 2010; **1 (1) (no pagination)**(7).
28. Weber JA, et al. The microRNA spectrum in 12 body fluids. *Clin Chem* 2010; **56**(11): 1733-41.
29. Wu F, et al. Exploration of microRNA profiles in human colostrum. *Annals of Translational Medicine* 2020; **8**(18): 13.
30. Zhou Y, et al. Exosomal circRNAs contribute to intestinal development via the VEGF signalling pathway in human term and preterm colostrum. *Aging (Albany NY)* 2021; **13**.
